# Dissecting the role of SEPHS1 in shaping an immunosuppressive microenvironment to promote tumor progression

**DOI:** 10.1007/s00262-025-04253-3

**Published:** 2025-12-24

**Authors:** Yunqing Liu, Yaxin Cheng, Wenxi Ji, Xiaotian Yuan, Guanjun Chen, Yiqi Tan, YuJie Cui, Yulong Huang, Zhijie Gao, Ke Cao

**Affiliations:** 1 Department of Oncology, Third Xiangya Hospital of Central South University, Changsha, 410013 China; 2 Department of Otolaryngology Head and Neck Surgery, Xiangya Hospital of Central South University, Changsha, 410008 China; 3 Postdoctoral Station of Clinical Medicine, The Third Xiangya Hospital, Central South University, Changsha, 410013 China

**Keywords:** SEPHS1, Selenium metabolism, Tumor immune evasion, CD8⁺ T cell infiltration, Immunotherapy sensitization

## Abstract

**Background:**

Cancer immunotherapy has revolutionized the treatment landscape for multiple malignancies, particularly melanoma. However, therapeutic resistance remains common, highlighting the need to identify novel regulators of antitumor immunity. Selenium is an essential micronutrient that modulates redox homeostasis and immune function through its incorporation into selenoproteins. Yet, the immunological roles of selenium metabolism-related enzymes, especially Selenophosphate Synthetase 1 (SEPHS1), remain poorly defined.

**Methods:**

We performed a comprehensive pan-cancer analysis using TCGA, CCLE, CPTAC, and cBioPortal datasets to evaluate the expression patterns and clinical relevance of selenium metabolism-related genes. A SELENOAMINO Score (SAS) was established to quantify pathway activity and explore its association with prognosis and tumor immune features. CRISPR-Cas9 functional screening data were integrated to identify selenium metabolism genes linked to immunotherapy response. SEPHS1 was further investigated in melanoma through in vitro and in vivo experiments, including gene knockdown, T cell co-culture, flow cytometry, and transcriptomic profiling.

**Results:**

Selenium metabolism-related genes exhibited heterogeneous expression and prognostic associations across cancers. SAS correlated with immune infiltration and clinical outcomes, suggesting an immunoregulatory role of selenium metabolism. SEPHS1 was frequently overexpressed and associated with poor prognosis, driven by promoter hypomethylation and copy number amplification. In melanoma, high SEPHS1 expression was linked to reduced CD8⁺ T cell infiltration and activation of immunosuppressive pathways. Knockdown of SEPHS1 enhanced CD8⁺ T cell recruitment and effector function, upregulated CXCL9/10, and significantly improved the therapeutic efficacy of anti-PD-1 blockade.

**Conclusions:**

SEPHS1 promotes immune evasion in melanoma by suppressing chemokines and limiting CD8⁺ T cell infiltration. Targeting SEPHS1 restores immune activity and potentiates immune checkpoint blockade, suggesting a novel immunometabolic strategy to enhance cancer immunotherapy.

**Supplementary Information:**

The online version contains supplementary material available at 10.1007/s00262-025-04253-3.

## Introduction

Cancer immunotherapy has emerged as one of the most transformative advances in oncology, offering durable clinical benefits across a broad range of malignancies. Among various immunotherapeutic modalities, immune checkpoint inhibitors (ICIs) targeting PD-1/PD-L1 and CTLA-4 have achieved unprecedented success in tumors such as melanoma, non-small cell lung cancer, and renal cell carcinoma [1–4]. By reinvigorating exhausted T cells and restoring antitumor immunity, ICIs have shifted the treatment paradigm from cytotoxicity-based approaches to immune-mediated tumor eradication. Melanoma, characterized by high tumor mutational burden and strong immunogenicity, has served as a prototypical model for understanding ICI efficacy and resistance mechanisms [5]. Despite these advances, the clinical benefit of ICB remains limited to a subset of patients, with both primary and acquired resistance frequently observed [6]. Resistance to immunotherapy arises from a complex interplay of tumor-intrinsic alterations and extrinsic immune regulatory networks within the tumor microenvironment (TME). Mechanistically, immune evasion can occur through impaired antigen presentation, upregulation of inhibitory ligands such as PD-L1, and exclusion of effector T cells from the tumor core [7]. In addition, emerging evidence highlights the role of cellular metabolism in shaping the immunosuppressive TME, thereby modulating therapeutic responsiveness [8–10].

Metabolic reprogramming is a hallmark of cancer, enabling tumor cells to meet the demands of rapid proliferation while concurrently influencing immune cell function [11]. Beyond classic metabolic pathways such as glycolysis, fatty acid synthesis, and glutamine metabolism, trace element metabolism—particularly involving iron, zinc, copper has recently been implicated in regulating antitumor immunity [12–18]. Selenium, an essential micronutrient, functions primarily through its incorporation into selenoproteins, which are critical for redox balance, immune regulation, and cell survival [19]. Clinical studies have associated selenium status with cancer risk and prognosis in a context-dependent manner [20, 21]. However, how selenium metabolism influences tumor immunity, especially in immunogenic tumors such as melanoma, remains largely unexplored.

Selenium is found in both inorganic forms, such as selenate and selenite, and organic forms, including selenomethionine and selenocysteine. Among these, selenocysteine represents the biologically active form incorporated into selenoproteins via a complex biosynthetic pathway [22]. The synthesis of selenophosphate, which serves as a key selenium donor, is catalyzed by the enzymes selenophosphate synthetase 1 (SEPHS1) and selenophosphate synthetase 2 (SEPHS2). While SEPHS2 is known to possess enzymatic activity essential for de novo selenocysteine biosynthesis, the role of SEPHS1 remains controversial. SEPHS1 is thought to lack direct catalytic activity but may participate in the stabilization and regulation of the SEPHS2 complex [19, 23]. Nevertheless, the contribution of SEPHS1 to cancer progression and immune regulation has not been systematically studied.

To investigate the potential link between selenium metabolism and tumor immunity, we first performed a pan-cancer analysis of selenium metabolism-related genes and established a selenium metabolism–related signature score (SELENOAMINO score, SAS) to quantify pathway activity across 33 tumor types. This screening revealed strong associations between selenium metabolism, immune infiltration, immune functional modules, and regulatory pathways, suggesting its involvement in immunoregulation. By integrating these findings with CRISPR-Cas9 screens identifying immune checkpoint regulators [24–26], SEPHS1 emerged as the top candidate directly identified as a sensitizer to anti-PD-1 therapy. We therefore focused on SEPHS1 for mechanistic characterization and functional validation. In this study, we demonstrate that SEPHS1 suppresses CXCL9 and CXCL10 expression, impairs CD8⁺ T cell infiltration, and promotes immune evasion in melanoma. Integrating pan-cancer, CRISPR, and immunotherapy cohort analyses, we identify SEPHS1 as a selenium metabolism-associated gene implicated in tumor immune regulation. Using in *vitro* and in *vivo* models, we further validate that SEPHS1 inhibition enhances anti-PD-1 efficacy. Collectively, this work establishes a previously unrecognized connection between selenium metabolism and antitumor immunity and provides a rationale for targeting SEPHS1 to overcome immunotherapy resistance.

## Methods and materials

### Pan-cancer data collection and processing

Expression patterns of SEPHS1 across cancer cell lines were evaluated using the Cancer Cell Line Encyclopedia (CCLE) [27]. Pan-cancer mRNA expression profiles, clinical data, survival outcomes, somatic mutations, copy number variation (CNV), and DNA methylation data (HumanMethylation450K) were obtained from the UCSC Xena platform (http://xena.ucsc.edu/), with raw data derived from The Cancer Genome Atlas (TCGA) and the Genotype-Tissue Expression (GTEx) project. Protein-level expression data were accessed from the Clinical Proteomic Tumor Analysis Consortium (CPTAC) [28].

Single-cell expression of SEPHS1 in distinct cell populations was assessed with the Tumor Immune Single-cell Hub (TISCH) [29], which enables visualization of gene expression across various tumor types and tumor microenvironment cell subsets. Additionally, single-cell RNA sequencing datasets GSE210347 and GSE215120 were downloaded from the Gene Expression Omnibus (GEO) and processed using the Seurat package. Cells with more than 15% mitochondrial content or fewer than 300 detected genes were excluded. Expression data were normalized using the log_2_(TPM + 1) method. Highly variable genes were selected, and principal component analysis (PCA) was performed using the top 15 components. Clustering was conducted with a resolution of 0.1 and visualized via Uniform Manifold Approximation and Projection (UMAP), followed by annotation using canonical marker genes.

Melanoma immunotherapy cohorts were obtained from PRJEB23709 (Gide et al.) in the TIGER database [30], and from GSE78220 (Hugo et al.) and GSE91061 (Riaz et al.) in GEO. Urothelial carcinoma immunotherapy cohorts were derived from IMvigor210 (Mariathasan et al.) in the IMvigor210CoreBiologies dataset and GSE176307 (Rose et al.) in GEO [31–34]. RNA sequencing data were preprocessed to remove low-quality samples and low-abundance genes, followed by normalization using log_2_(TPM + 1). A complete list of the abbreviations and full names of the 33 cancer types analyzed in this study is provided in Table S1.

### Calculation of SELENOAMINO score

To evaluate the transcriptional pattern of selenium metabolism across cancers, we constructed a selenium metabolism–related signature score (SELENOAMINO score, SAS) using single-sample gene set enrichment analysis (ssGSEA). ssGSEA was performed with the GSVA R package using the “ssgsea” algorithm and default parameters to calculate enrichment scores for each sample. The gene set was obtained from the MsigDB “KEGG_SELENOAMINO_ACID_METABOLISM.v2024.1.Hs”, which contains 26 human genes involved in selenoamino acid metabolism (Table S2). The resulting enrichment scores were normalized by z-score transformation and subsequently applied for correlation and visualization analyses.

### Genomic alterations and instability analysis

Mutation frequencies were examined in cBioPortal. The “Cancer Type Summary” module assessed five alteration types: mutations, amplifications, shallow deletions, deep deletions, and structural variants [35, 36]. Differences in *SEPHS1* expression across CNV categories were analyzed by Kruskal–Wallis tests. Somatic mutations were parsed using “maftools”. Correlations between *SEPHS1* expression and genomic instability metrics (aneuploidy, homologous recombination defects, tumor ploidy, SNV neoantigens, nonsilent and silent mutation rates) were calculated with cor.test and visualized with the “fmsb” package.

### Survival analysis

To assess the prognostic relevance of *SEPHS1* expression, we analyzed overall survival (OS), disease-specific survival (DSS), disease-free interval (DFI), and progression-free interval (PFI) across cancer types. Kaplan–Meier analysis was performed using the survival and “survminer” R packages, with optimal expression cutoffs and a minimum group ratio of 0.2. Significance was evaluated using the log-rank test. Univariate Cox regression was also conducted to calculate hazard ratios (HRs) and 95% confidence intervals (CIs). Results were visualized using the “ComplexHeatmap” package.

### Correlation analysis of mRNA expression, DNA methylation, and RNA modification patterns

Pan-cancer DNA methylation data were annotated using the “ChAMP” R package. Spearman correlations between *SEPHS1* expression and DNA methylation beta values at various genomic loci were calculated using the cor.test function. RNA modification-related genes and annotations were obtained from the RM2Target database (http://rm2target.canceromics.org/#/home), and their expression levels were analyzed for correlation with *SEPHS1* expression.

### Functional analysis of SEPHS1 in tumor immune regulation using CRISPR screening data

The immunoregulatory role of SEPHS1 was assessed using the ICRAFT database (https://icraft.pku-genomics.org/#/homepage), which compiles 558 CRISPR screening datasets, two million single-cell RNA-seq profiles, and clinical data from 943 immunotherapy-treated patients [37]. Within the “Gene Exploration” module, we focused on three major screening categories: “Sorting by marker expression”, “Co-culture with immune cells” and “In vivo CRISPR screens.” All datasets were analyzed following the standard ICRAFT pipeline, providing functional evidence for SEPHS1 involvement in multiple immune regulatory processes and supporting its potential as an immunotherapeutic target.

### Immune microenvironment correlation analysis

The correlation between SEPHS1 mRNA expression and immune checkpoint genes as well as immune cell infiltration levels across multiple cancer types was systematically analyzed using the “TCGAplot” R package. Additionally, the association between *SEPHS1* expression and tumor mutation burden (TMB) was evaluated. The Tumor Immune Estimation Resource (TIMER) database was further used to assess the correlation between *SEPHS1* expression and the infiltration level of myeloid-derived suppressor cells (MDSCs).

### Cancer-immunity cycle scoring

The Tracking Tumor Immunophenotype (TIP) database was utilized to systematically evaluate the immune status of tumors and the proportion of infiltrating immune cells involved in each step of the cancer-immunity cycle. Based on RNA-seq or microarray data, TIP provides quantitative scores for seven steps of the cancer-immunity cycle: (1) release of cancer cell antigens; (2) cancer antigen presentation; (3) priming and activation of immune cells; (4) trafficking of immune cells to tumors; (5) infiltration of immune cells into tumors; (6) recognition of cancer cells by T cells; and (7) killing of cancer cells by T cells. For each tumor sample, quantitative scores for these steps were extracted to reflect the level of immune activity within the tumor microenvironment. Spearman correlation analysis was used to evaluate the association between *SEPHS1* expression and each TIP step score. Correlations among the TIP steps themselves were also assessed. Visualization was performed using the “linkET” R package to generate correlation heatmaps depicting the relationship between *SEPHS1* expression and dynamic immune processes.

### Immune signature scoring

Immune-related gene signatures were analyzed, with several gene sets derived from previously published studies [38]. These immune-related signatures comprehensively capture the immune landscape across multiple cancer types, encompassing immune cell populations, immune functional modules, and regulatory pathways. Specifically, they include signatures reflecting major immune phenotypes such as the six pan-cancer immune subtypes, distinct patterns of immune-cell infiltration, cytokine and interferon signaling, wound-healing and TGF-β activity, and antigen-presentation machinery, thereby enabling systematic evaluation of tumor immune microenvironmental heterogeneity. Cytolytic activity (CYT) was assessed based on the transcript levels of two key cytolytic effectors, Granzyme A (GZMA) and Perforin (PRF1), to evaluate immune effector activity within solid tumors and estimate the combined cytotoxic potential of CD8⁺ T cells and natural killer (NK) cells [39]. For MeTIL signature analysis, principal component analysis (PCA) was performed on individual methylation beta values of MeTIL marker genes to generate a composite MeTIL score. The resulting scores were standardized using the Z-score transformation formula (x-μ)/σ. Samples were divided into high- and low-expression groups based on the median *SEPHS1* expression, and differences in MeTIL scores between the two groups were evaluated using the Wilcoxon rank-sum test.

### Cell culture and transfection

Murine bladder cancer MB49, melanoma B16F10, and human embryonic kidney HEK293T cell lines were obtained from the Cell Bank of the Chinese Academy of Sciences (Shanghai, China). MB49, B16F10 and HEK293T cells were cultured in DMEM (Pricella, cat#PM150210). supplemented with 10% heat-inactivated FBS (Pricella, cat#164210–50), 100 U/mL penicillin–streptomycin, and maintained at 37 °C in a humidified incubator with 5% CO₂. shRNA plasmids targeting Sephs1 were constructed by GeneChem Co., Ltd. (Shanghai, China). The target sequences of Sephs1 shRNAs were as follows (5′-3′): *Sephs1*-sh1: 5′-GCCTCATGCACACGTTCAATG-3′, *Sephs1*-sh2: 5′-GGTTCTGTGCAGAGATCAAGT-3′, *Sephs1*-sh3: 5′-GCAGGATAGCATGTGCCAATG-3′. These sequences were cloned into the pLKO.1-LUC-PURO vector using AgeI and EcoRI restriction sites. Plasmids were packaged in HEK293T cells, and lentiviral transduction was performed using jetPRIME® transfection reagent (Polyplus, cat#101000046).

### CCK-8 assay

MB49 and B16F10 cells were seeded into 96-well plates at a density of 2000 cells per well in 100 μL of complete medium. At 12, 24, 48, and 72 h after seeding, 10 μL of CCK-8 reagent (Biosharp Life Sciences, cat#BS350A) was added to each well and incubated at 37 °C for 2 h. The optical density (OD) at 450 nm was measured using a microplate reader to evaluate cell proliferation.

### Colony formation assay

MB49 and B16F10 cells were plated in 6-well plates at a density of 800 cells per well and cultured for 1–2 weeks until visible colonies formed. Only colonies consisting of more than 50 cells were considered and counted under a light microscope. Colonies were fixed with 4% paraformaldehyde, stained with 0.1% crystal violet, and imaged.

### Transwell matrigel invasion assay

Transwell chambers were pre-coated with Matrigel to assess the invasive capacity of *Sephs1* knockdown and control MB49 and B16F10 cells. Matrigel was diluted in serum-free medium, mixed thoroughly, and 50 μL of the mixture was added to each insert, followed by incubation in a CO_2_ incubator for 2 h. Afterward, 100 μL of serum-free medium was added to each insert and incubated for an additional 30 min. Cells were washed with PBS and resuspended in serum-free DMEM. For invasion, 200 μL of the cell suspension was seeded into the upper chamber, while 600 μL of DMEM containing 20% FBS was added to the lower chamber as a chemoattractant. After 24 h of incubation, non-invading cells on the upper surface of the membrane were gently removed. Invading cells on the lower surface were fixed, stained with 0.1% crystal violet, and washed. The number of stained cells was counted in three randomly selected fields under a microscope.

### Wound-healing assay

Cells were cultured in 6-well plates until they formed a 100% confluent monolayer. A sterile 10 μL pipette tip was used to create a straight scratch in the cell monolayer. The medium was then replaced with serum-free medium after washing to remove debris. Wound closure was monitored by capturing images at 0 and 48 h.

### Animal experiments

Male C57BL/6J mice aged 6–8 weeks were purchased from SJA Laboratory Animal Co., Ltd. (Changsha, China). All animal procedures were approved by the Institutional Animal Care and Use Committee of Central South University (Approval No. CSU-2024-0256). For tumor implantation, MB49 bladder cancer cells (2 × 10^6^ cells in 200 μL PBS) or B16F10 melanoma cells (1 × 10^6^ cells in 200 μL PBS) with stable gene expression were subcutaneously injected into the flank region of each mouse. Tumor growth was monitored every three days starting from one week post-injection. All animal experiments were conducted in a blinded manner, with mice coded using three-digit identifiers to conceal group allocation. For in vivo treatment, mice bearing B16F10 tumors received intraperitoneal injections of anti-PD-1 antibody (Bioxcell, cat#BE0146) or its IgG2a isotype control (Bioxcell, cat#BE0089) at a dose of 10 mg/kg, administered every three days for a total of four doses.

### T cell–mediated tumor cell killing and co-culture assay

Peripheral blood mononuclear cells (PBMCs) were isolated from the spleens of B16F10 tumor-bearing C57BL/6J mice by mechanical dissociation through a nylon mesh followed by density gradient centrifugation (800 g, 30 min). CD8⁺ T cells were sorted from PBMCs using flow cytometry and activated in complete DMEM supplemented with recombinant murine IL-2 (PeproTech, cat#212-12-1MG) and ImmunoCult Mouse CD3/CD28/CD2 T Cell Activator (STEMCELL Technologies, cat#100-1572) for 7 days, with periodic media replenishment to maintain cell viability and density (< 1 × 10^6^ cells/mL). For co-culture, B16F10 cells were seeded in 96-well plates (1 × 10^5^ cells/well), allowed to adhere overnight, and then co-incubated with activated CD8⁺ T cells at an effector-to-target ratio of 10:1 for 24 h. Tumor cell viability was assessed by crystal violet staining (absorbance at 590 nm) and flow cytometry using Annexin V-FITC/PI to quantify apoptotic cells. All conditions were tested in triplicate with appropriate negative and blank controls.

### T cell chemotaxis assay

The chemotactic response of CD8⁺ T cells was evaluated using a 24-well Transwell system with 5 μm pore size inserts. CD8⁺ T cells were isolated from mouse PBMCs via flow cytometric sorting and resuspended at a concentration of 1 × 10^5^ cells in 200 μL RPMI medium. The cells were added to the upper chambers, while 600 μL of conditioned media collected from different stably transduced tumor cell lines was placed in the lower chambers. After incubation at 37 °C for 3 h, the cells that had migrated into the lower chamber were collected and quantified by flow cytometry.

### Flow cytometric analysis of tumor-infiltrating T cell subsets and effector function

Subcutaneous tumors were excised from C57BL/6 J tumor bearing mice and mechanically dissociated in pre chilled PBS to obtain single cell suspensions. Cells were adjusted to a concentration of ≤ 1 × 10^7^ cells/mL and aliquoted at 100 μL per tube for multiparametric flow cytometry using the Cytek Northern Lights cytometer. Viability staining was performed with Zombie Aqua Fixable Viability Kit (BioLegend, Cat#423101) at 4 °C for 15 min. After washing, cells were incubated with Fc Block (BD Biosciences, Cat#553141) at room temperature for 10 min to prevent nonspecific binding. Surface staining was conducted in the dark at 37 °C for 30 min using the following antibodies: CD45 (BD Biosciences, Cat#557659), CD11b (BD Biosciences, Cat#562128), CD3 (eBioscience, Cat#14–0032-82), CD4 (BD Biosciences, Cat#557307), and CD8α (BD Biosciences, Cat#562283). Cells were then fixed and permeabilized using the BD Pharmingen Transcription Factor Buffer Set and stained intracellularly at 37 °C for 30 min with Ki-67 (BD Biosciences, Cat#566172), IFN-γ (BioLegend, Cat#505836), and Granzyme B (eBioscience, Cat#12-8899-41). All antibodies were used following manufacturer instructions. Fluorescence minus one (FMO) controls and single stain controls were included for compensation adjustment and validation of antibody specificity. Cells were resuspended in PBS at 1 × 10^6^ cells/mL and analyzed on the flow cytometer. FlowJo v10.8 software was used to quantify T cell populations (CD3^+^CD4⁺ and CD3^+^CD8⁺), proliferating cells (Ki-67^+^), and effector markers (Granzyme B^+^ and IFN-γ^+^).

### Quantitative real-time PCR (RT-qPCR)

Total RNA was extracted using the Steadypure Quick RNA Extraction Kit (Accurate Biology, cat#AG21023), and RNA purity was assessed by spectrophotometry (A260/A280>1.8). One microgram of RNA was reverse-transcribed into cDNA using HiScript II Q RT SuperMix (Vazyme, cat#R223). RT-qPCR was performed using SYBR Green Master Mix (Vazyme, cat#Q711) on a real-time PCR system. Relative gene expression was calculated using the 2^−ΔΔCt^ method, with GAPDH as the internal reference gene. Primer sequences used for target genes are listed in Table S3.

### Western blotting

Western blotting was performed using protein lysates from *Sephs1* knockdown and control MB49 or B16F10 cells. Total protein was extracted using RIPA buffer supplemented with protease and phosphatase inhibitors, followed by centrifugation at 12,000 rpm for 15 min at 4 °C. Protein concentration was determined by the BCA assay. Equal amounts of denatured protein were separated by SDS-PAGE and transferred onto PVDF membranes. After blocking, membranes were incubated overnight at 4 °C with a primary antibody against SEPHS1 (Santa Cruz Biotechnology, cat#sc-365945), followed by incubation with HRP-conjugated secondary antibodies. Protein bands were detected using enhanced chemiluminescence (ECL) reagents.

### Enzyme-linked immunosorbent assay (ELISA)

Cell culture supernatant was collected by centrifugation at 1000 × g for 20 min. ELISA was conducted following manufacturer instruction. In brief, 100 μL of standard or sample was added to each well of a pre-coated plate, followed by incubation with biotinylated antibody, HRP-conjugated reagent, and TMB substrate. After addition of stop solution, absorbance was measured at 450 nm. The kits used were Mouse MIG (Cxcl9) ELISA Kit (ELK Biotechnology, cat#ELK1336) and Mouse IP10 (Cxcl10) ELISA Kit (ELK Biotechnology, cat#ELK1279).

### Immunohistochemistry (IHC) and multiplex immunohistochemistry (mIHC)

Melanoma tissue microarrays were commercially constructed by ZhuoLi Biotech Co., Ltd. (Shanghai, China), while paraffin-embedded tumor tissue sections from clinical samples were collected with informed consent from all patients and approved by the institutional ethics committee. Sections were deparaffinized in xylene, rehydrated through graded ethanol, and subjected to antigen retrieval using EDTA buffer under microwave heating. For IHC, endogenous peroxidase activity was blocked with 3% H₂O₂, followed by blocking with 5% BSA for 30 min. Primary antibodies (Ki67, Proteintech, cat#27309-1-AP; CD8a, Proteintech, cat#29896-1-AP; SEPHS1, Santa Cruz Biotechnology, cat#sc-365945) were incubated overnight at 4 °C. After washing, HRP-conjugated secondary antibodies were added for 30 min at room temperature, and signals were developed using DAB substrate. Sections were counterstained with hematoxylin, dehydrated, and mounted for imaging. For mIHC, after sequential rounds of primary antibody incubation, HRP-conjugated secondary antibody reaction, and fluorophore development using TSA amplification, antibodies were stripped using preheated stripping buffer at 37 °C. This cycle was repeated for multiplex staining with different markers. Nuclei were counterstained with DAPI, and autofluorescence was quenched before mounting with anti-fade reagent. Images were captured using the Pannoramic MIDI scanner (3DHISTECH).

### Statistical analysis

All statistical analyses and data visualization were performed using R (v4.3.2) and GraphPad Prism (v.9.2) Experimental data were obtained from at least three independent replicates and are presented as mean ± standard error of the mean. For comparisons between two groups, an unpaired Student’s t-test was used for normally distributed data, while the Mann–Whitney U test was applied for non-normal distributions. One-way ANOVA followed by Tukey’s post hoc test was used for multiple group comparisons. Differences in categorical variables were assessed using the Chi-square test. Correlations were evaluated using Spearman’s rank correlation coefficient. Statistical significance was indicated by asterisks unless otherwise specified: *p < 0.05, **p < 0.01, ***p < 0.001, and **** p < 0.0001.

## Results

### Pan-cancer analysis reveals expression patterns, epigenetic regulation, genomic alterations, and prognostic value of selenium metabolism-related genes

To characterize the expression and functional significance of selenium metabolism-related genes across cancers, we conducted a comprehensive pan-cancer analysis using TCGA datasets. Differential expression analysis across 23 cancer types with paired tumor and normal samples revealed substantial heterogeneity. Among the 26 selenium metabolism–related genes, 12 (46.2%) were upregulated in at least half of the analyzed cancer types, while 14 (53.8%) showed predominant downregulation (Table S4). Genes such as *BUD23, MARS1, METTL6, METTL2B, LCMT1, AHCY, SEPHS1,* and *SEPHS2* were consistently upregulated in multiple cancer types, suggesting a broad role in tumor progression, whereas *MAT2B, AHCYL1, AHCYL2, GGT7,* and *PAPSS2* were frequently downregulated, with *GGT6* showing pronounced suppression in renal cancers. Nevertheless, most of the selenium metabolism–related genes displayed cancer-type–specific expression patterns, indicating context-dependent regulation (Figure S1). Genomic alteration profiling revealed frequent single nucleotide variants (SNVs) and somatic copy number alterations (SCNAs), particularly in colon adenocarcinoma, skin cutaneous melanoma, and uterine corpus endometrial carcinoma, where nearly all studied genes showed varying degrees of mutations. *PAPSS1* and *PAPSS2* exhibited high mutation frequencies across more than 10 cancer types, with *PAPSS1* mutated in 5.8% of UCEC cases (Figure S2A-B). SCNAs were common across most cancer types (Figure S2C), and copy number gains showed strong positive correlations with mRNA expression, indicating that genomic alterations may drive transcriptional activation (Figure S2D). Survival analysis using univariate Cox regression demonstrated that the prognostic value of selenium metabolism-related genes varied considerably among cancer types (Figure S3A-D). For example, *MARS1* was a significant risk factor in at least 10 cancers, whereas *LCMT1* and *LCMT2* were protective in several tumors, especially renal cancers. *SEPHS1* displayed a dual prognostic role, acting as protective in gliomas and hematological malignancies but adverse in liver cancer and melanoma, suggesting tumor-specific functions.

### Correlation between selenium metabolism activity and immune-related features

**Fig. 1:**
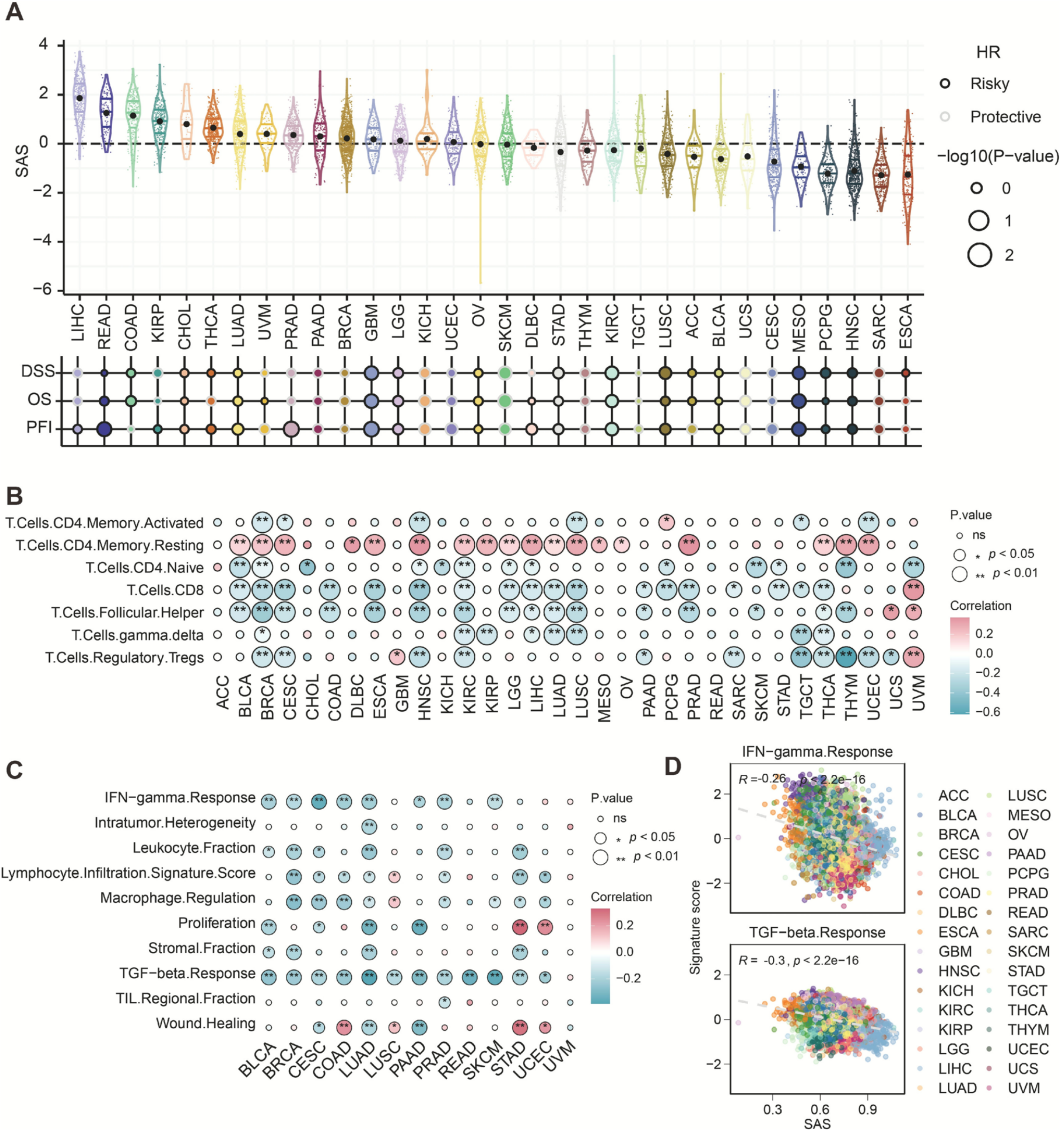
Prognostic value and immune landscape of the SAS score across pan-cancer. **A** Distribution of the SAS score across various cancer types and its association with OS, DSS, and PFI. The size of each circle represents the -log10(p-value), with black outlines indicating risk factors (Hazard ratio > 1) and white outlines indicating protective factors (Hazard ratio < 1). **B** Spearman correlation analysis between the SAS score and infiltration levels of different T cell subpopulations. Dot color represents the correlation coefficient, and dot size indicates statistical significance (ns: not significant, *p < 0.05, **p < 0.01). **C** Correlations between the SAS score and various immune-related features, including immune functional pathways, tumor immune heterogeneity, leukocyte fraction, macrophage regulation, stromal content, and regional distribution of tumor-infiltrating lymphocytes (TILs). **D** Scatter plots showing the association between the SAS score and IFN-γ response (top) and TGF-β response (bottom), respectively, across cancer types. Pearson correlation coefficients and corresponding p-values are shown

To evaluate the immunological relevance of selenium metabolism, we constructed a SAS score using ssGSEA based on the expression of selenium metabolism-related genes. The SAS score showed variable distribution across cancer types, with higher levels observed in gastrointestinal tumors such as hepatocellular carcinoma, rectal adenocarcinoma, and colon adenocarcinoma, and lower levels in sarcoma, esophageal carcinoma, and acute myeloid leukemia (Fig. 1A). SAS was also differentially associated with prognosis, including OS, DSS, and PFI, suggesting cancer-type-specific prognostic implications of selenium metabolism.

To explore the relationship between selenium metabolism activity and the tumor immune landscape, we assessed the correlation between SAS and immune cell infiltration. Across the TCGA pan-cancer cohort, SAS was negatively correlated with infiltration levels of multiple T cell subsets, particularly CD8⁺ T cells, indicating a potential immunosuppressive role of selenium metabolism. Interestingly, SAS showed a positive correlation with resting memory CD4⁺ T cells, suggesting a complex, context-dependent immune regulatory effect (Fig. 1B).

Further analysis revealed that SAS was significantly negatively correlated with multiple immune-related pathways and features, including leukocyte fraction, cell proliferation, wound healing, macrophage regulation, lymphocyte infiltration, and cytokine response signatures. Notably, SAS showed strong negative correlations with both IFN-γ and TGF-β response pathways (Fig. 1C). Across 32 tumor types, SAS was moderately negatively correlated with IFN-γ response (R = − 0.25, *p* < 2.2e−16) and more strongly with TGF-β response (R = − 0.30, *p* < 2.2e−16) (Fig. 1D).

Collectively, these findings suggest that elevated selenium metabolism activity may contribute to the formation of an immunosuppressive tumor microenvironment by suppressing key immune effector pathways. This metabolic state may promote a “cold tumor” phenotype and potentially impair response to immunotherapy.

### Functional inference of SEPHS1 in tumor immune regulation based on CRISPR screening data

Given the observed association between selenium metabolism and tumor immunity, we integrated previously published in vivo CRISPR screening datasets related to immune checkpoint blockade or anti-PD-1 therapy [24–26, 40]. Candidate genes were extracted from curated libraries focusing on kinases, phosphatases, surface markers, antigen presentation, immune processes, and chromatin remodeling, and were screened using murine tumor models including melanoma, colorectal, pancreatic, lung, and renal cancers. To identify immune-dependent regulators, we prioritized cohorts comparing immune-competent or immunotherapy-treated mice with their immunodeficient counterparts and applied a threshold of FDR ≤ 0.05 (Table S5). We specifically focused on identifying genes whose loss conferred immune escape or resistance to immunotherapy.These included *H2-T23*, *TAP1*, *TAP2*, and *ERAP1*, which are involved in antigen processing and presentation and are known to be IFN-γ-inducible and associated with resistance to immune checkpoint blockade. In addition, *TRAF3* has been shown to enhance MHC-I expression and sensitize tumors to T cell killing and ICB therapy, while *SOCS1* loss has been reported to improve adoptive cell transfer efficacy [41–43].

**Fig. 2:**
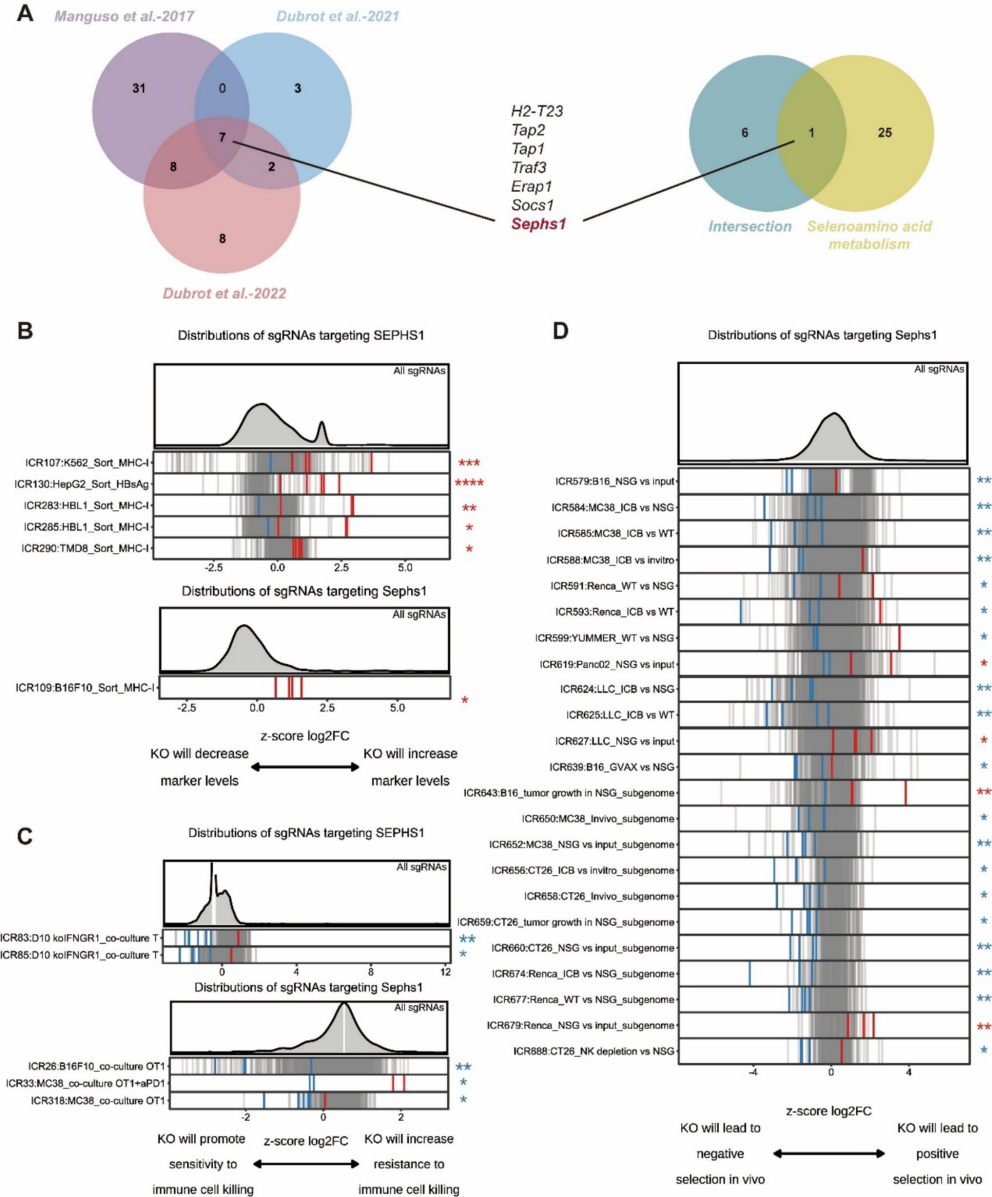
Identification of SEPHS1 as a Regulator of Tumor Immune Evasion via CRISPR Screens. **A** Venn diagrams summarizing CRISPR screening intersections. Left: overlap of immune escape-related genes identified in three independent CRISPR screens, with SEPHS1 among the common hits. Right: intersection between immune escape-related genes and selenoamino acid metabolism-related gene sets, highlighting SEPHS1 as the sole overlapping gene. **B** Rank plots showing the distribution of sgRNAs targeting SEPHS1/Sephs1 in the “marker-based sorting” category. Data are shown for both human and murine cancer cell lines, with changes in MHC-I expression after gene knockout represented as Z-score of log₂ fold change (log₂FC). A positive log₂FC indicates increased marker expression upon SEPHS1 knockout. **C** sgRNA distribution of SEPHS1/Sephs1 in the “immune co-culture” screening category. Human and mouse cancer cells were co-cultured with immune effector cells under various conditions. Changes in immune-mediated killing sensitivity following knockout are indicated by Z-score log₂FC, with positive values reflecting resistance to killing and negative values indicating sensitization. **D** In vivo CRISPR screening results for Sephs1 in different immunological contexts, including wild-type and immunodeficient mice, as well as anti-PD-1 treatment. Z-score log₂FC values indicate changes in in vivo selection pressure after *Sephs1* knockout, with negative values suggesting depletion and positive values indicating enrichment. The red and blue tick marks denote datasets in which the corresponding gene was identified as significantly depleted (red) or enriched (blue) under the indicated immune context. *FDR ≤ 0.25, **FDR ≤ 0.1, ***FDR ≤ 0.05, ****FDR ≤ 0.01

Cross-referencing these seven immune regulators with the selenoamino acid metabolism gene set identified SEPHS1 as the only overlapping gene (Fig. 2A). As the immunoregulatory role of SEPHS1 has not been previously characterized, we focused on investigating its potential functions in modulating antitumor immunity. Utilizing the ICRAFT-integrated CRISPR screening platform, we systematically assessed the impact of SEPHS1 on antigen presentation, T cell activation, and immune responsiveness. In both human and murine cancer cell lines, marker-based CRISPR screening showed that SEPHS1/Sephs1 knockout significantly increased MHC-I expression, suggesting enhanced tumor immunogenicity (Fig. 2B). In co-culture models using tumor cells and T cells, sgRNAs targeting SEPHS1/Sephs1 were enriched in negatively selected populations, indicating that SEPHS1 loss sensitized tumor cells to T cell-mediated killing (Fig. 2C). Furthermore, in vivo CRISPR screens under various immune contexts revealed that Sephs1-deficient tumor cells were negatively selected, implying decreased fitness under immune pressure (Fig. 2D, Table S6).

Collectively, these findings demonstrate that SEPHS1 modulates multiple immunoregulatory processes including antigen presentation and T cell-mediated cytotoxicity, highlighting its potential as a therapeutic target to enhance response to immune checkpoint blockade.

### Expression landscape of SEPHS1 across cancer types and cell populations

**Fig. 3:**
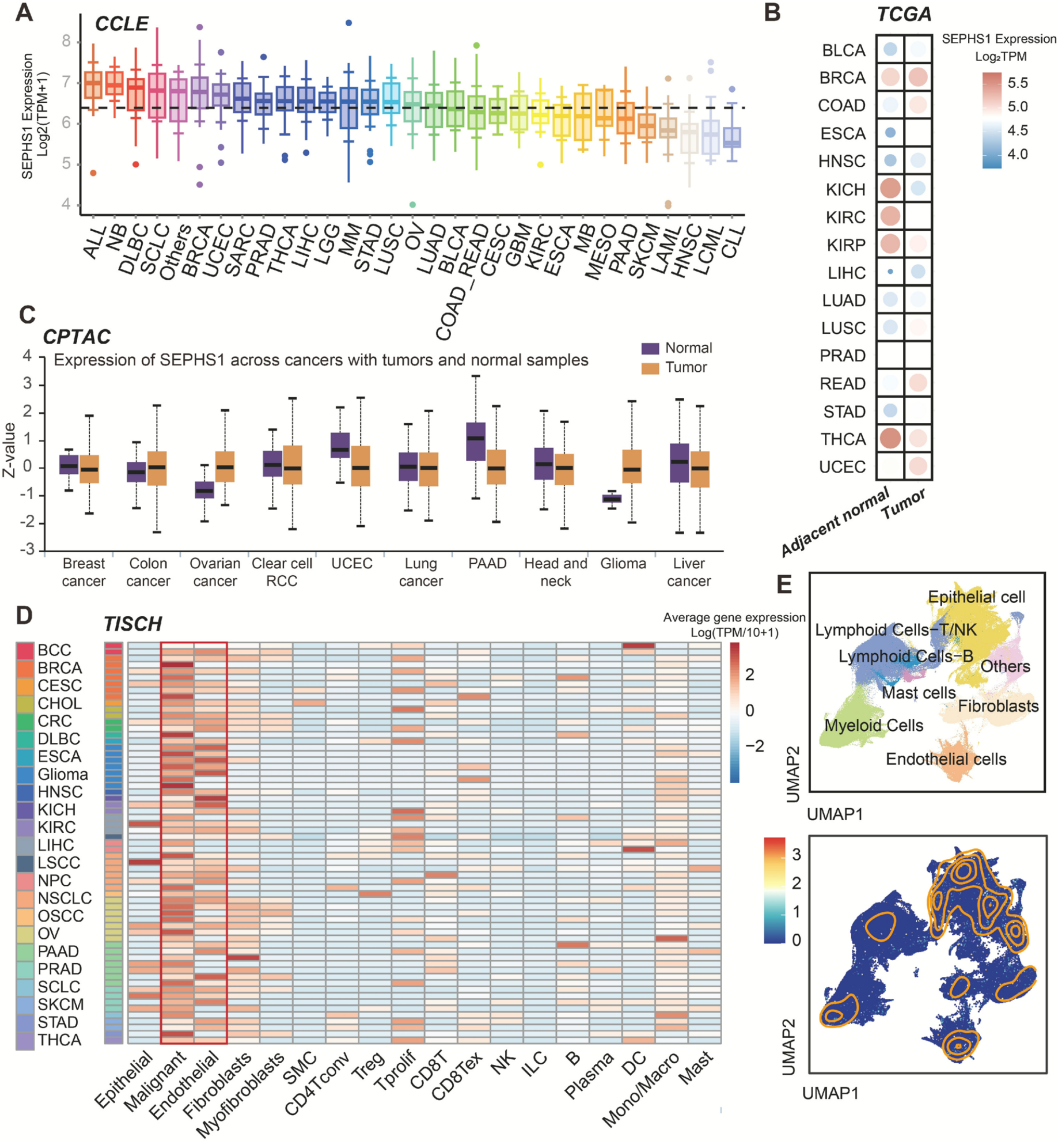
Expression landscape of SEPHS1 across pan-cancer datasets. **A** Boxplot showing the distribution of SEPHS1 mRNA expression levels in various cancer cell lines from the CCLE database. Colors indicate different cancer types (CCLE lineages). **B** Bubble heatmap illustrating SEPHS1 mRNA expression differences between tumor tissues and adjacent normal tissues across multiple cancer types in the TCGA cohort. Circle size represents expression level and color indicates relative magnitude. **C** Boxplots comparing SEPHS1 protein expression between tumor and normal tissues across cancer types in the CPTAC proteomic dataset. **D** Heatmap showing cell type specific SEPHS1 expression at the single-cell level across cancers based on the TISCH database. Major cell types include epithelial cells, endothelial cells, fibroblasts, lymphoid cells, and myeloid cells. **E** UMAP visualization presenting the two-dimensional distribution of SEPHS1-expressing cells in the pan-cancer single-cell atlas. Colors represent distinct cell clusters

To investigate the expression profile of *SEPHS1* across cancers, we first analyzed RNA-seq data from 1019 human cancer cell lines obtained from the CCLE. SEPHS1 showed the highest expression in acute lymphoblastic leukemia and neuroblastoma, and the lowest expression in chronic myeloid leukemia and chronic lymphocytic leukemia. Elevated expression was also observed in diffuse large B-cell lymphoma, small cell lung cancer, breast cancer, and endometrial cancer (Fig. 3A).

We next evaluated *SEPHS1* mRNA expression in tumor versus adjacent normal tissues across TCGA cancer types. *SEPHS1* was significantly upregulated in tumor tissues compared to normal tissues in most cancer types, suggesting a broad role in tumorigenesis (Fig. 3B). At the protein level, data from the CPTAC showed that SEPHS1 protein expression was significantly elevated in colon cancer, ovarian cancer, and glioblastoma compared to adjacent normal tissues, mirroring its mRNA expression pattern (Fig. 3C, Table S7).

To explore *SEPHS1* expression at the single-cell level, we analyzed data from TISCH, a tumor immune single-cell RNA-seq database. *SEPHS1* was highly expressed in epithelial and endothelial cells across multiple tumor types, with malignant epithelial cells showing higher expression than their normal counterparts. *SEPHS1* was also detectable in fibroblasts and immune cell subsets, particularly in proliferating T cells and monocyte/macrophage populations, suggesting potential involvement in immune regulation within the tumor microenvironment (Fig. 3D).

To validate these findings, we further analyzed single-cell RNA-seq datasets from GSE210347 and GSE215120. After normalization and dimensionality reduction using UMAP, *SEPHS1* expression was predominantly observed in epithelial cells, followed by endothelial cells, consistent with the TISCH-based results (Fig. 3E). These results support a potential role for SEPHS1 in driving tumor development across multiple cancer types.

### *SEPHS1* expression is associated with DNA methylation, RNA modifications, and genomic instability

To investigate the regulatory mechanisms underlying the differential expression of *SEPHS1* between tumors and adjacent normal tissues, we analyzed its association with DNA methylation and RNA modification patterns. Annotation of *SEPHS1* methylation probes revealed that promoter methylation was significantly negatively correlated with *SEPHS1* mRNA expression in most cancer types (Figure S4A). For instance, in skin cutaneous melanoma, the Spearman correlation coefficient between SEPHS1 promoter methylation and mRNA expression was − 0.33 (*p* = 1.34e−13), suggesting transcriptional repression by hypermethylation (Figure S4B). Comparative analysis of tumor versus normal tissues showed that enhancer and promoter-5′UTR regions of SEPHS1 exhibited significantly higher methylation levels in several cancers, including bladder urothelial carcinoma and invasive breast carcinoma, further supporting a potential repressive role of DNA methylation on *SEPHS1* transcription (Figure S4C).

In addition to epigenetic control at the DNA level, we explored post-transcriptional regulation via RNA modifications. Correlation analysis revealed that *SEPHS1* expression was broadly and positively associated with regulators of seven major RNA modification types across multiple cancers. These included writers, erasers, and readers of m^6^A, m^5^C, m^1^A, and other RNA marks, suggesting a coordinated post-transcriptional regulatory interplay. The consistent positive correlations across cancer types imply that RNA modification networks may cooperatively contribute to SEPHS1 upregulation in tumor cells (Figure S4D). Together, these findings indicate that *SEPHS1* expression is regulated by both DNA methylation and RNA modifications in a cancer type-specific manner.

To further explore *SEPHS1* at the genomic level, we analyzed its mutation landscape and association with genomic instability indicators. cBioPortal analysis showed that *SEPHS1* exhibited modest alteration alteration frequencies in uterine corpus endometrial carcinoma and bladder urothelial carcinoma, mainly due to point mutations and copy number amplification, respectively. Occasional structural variants were observed in cholangiocarcinoma (Figure S5A). Among five major alteration types—point mutation, amplification, deep deletion, shallow deletion, and structural variation amplification was the most prevalent (Figure S5B). Spearman correlation analysis demonstrated that *SEPHS1* expression was significantly positively correlated with multiple indicators of genomic instability, including aneuploidy score, homologous recombination deficiency (HRD), tumor ploidy, SNV neoantigen load, and both silent and non-silent mutation rates. High *SEPHS1* expression was consistently associated with elevated chromosomal instability in bladder, breast, and lung cancers (Figure S5C-H). These findings suggest that *SEPHS1* may contribute to or reflect genomic instability in tumors and could be linked to tumor progression and immune evasion.

### Prognostic relevance of *SEPHS1* expression across cancer types

**Fig. 4:**
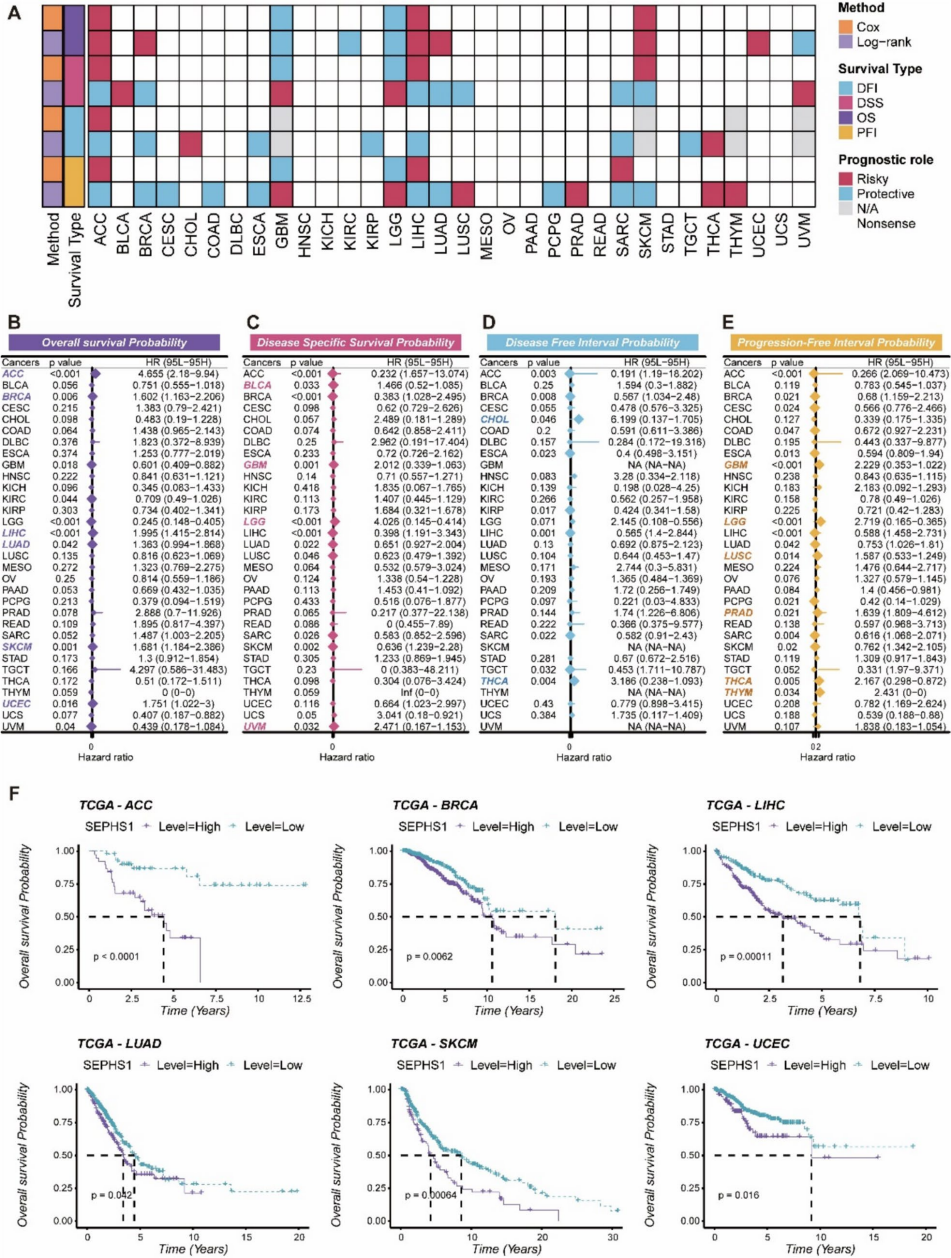
Prognostic analysis of SEPHS1 expression across pan-cancer types. **A** Heatmap showing the prognostic significance of SEPHS1 mRNA expression in different cancers evaluated by Cox regression and log-rank tests. Colors indicate the prognostic role as risk or protective factors. **B**–**E** Forest plots presenting the hazard ratios and 95% confidence intervals of SEPHS1 expression for OS, DSS, PFI, and DFI. Hazard ratios were calculated by univariate Cox regression. Significant results are highlighted. **F** Kaplan–Meier curves displaying the overall survival probability of SEPHS1 high and low expression groups in adrenocortical carcinoma, invasive breast carcinoma, liver hepatocellular carcinoma, lung adenocarcinoma, skin cutaneous melanoma, and uterine corpus endometrial carcinoma from the TCGA dataset. All analyses were performed using RNA-seq expression and clinical data obtained from the UCSC Xena platform

To assess the association between *SEPHS1* mRNA expression and patient prognosis, we performed Kaplan–Meier survival analyses combined with log-rank tests and univariate Cox regression models across multiple cancer types. All analyses were based on RNA-seq expression profiles and corresponding clinical data from the TCGA pan-cancer cohort obtained via the UCSC Xena platform. *SEPHS1* expression was significantly associated with survival outcomes, and high *SEPHS1* levels correlated with poor prognosis in several cancer types (Fig. 4A). Specifically, elevated *SEPHS1* expression was associated with reduced OS in adrenocortical carcinoma (HR = 3.462, *p* < 0.001), liver hepatocellular carcinoma (HR = 2.598, *p* < 0.001), and skin cutaneous melanoma (HR = 1.503, *p* < 0.001) (Fig. 4B-E). Kaplan–Meier curves based on TCGA data further confirmed that high SEPHS1 expression was linked to significantly lower OS probabilities in adrenocortical carcinoma, invasive breast carcinoma, liver hepatocellular carcinoma, lung adenocarcinoma, skin cutaneous melanoma, and uterine corpus endometrial carcinoma (Fig. 4F). These results suggest that SEPHS1 may serve as an unfavorable prognostic biomarker in multiple cancer types.

### SEPHS1 expression is associated with malignant phenotypes across cancers

**Fig. 5:**
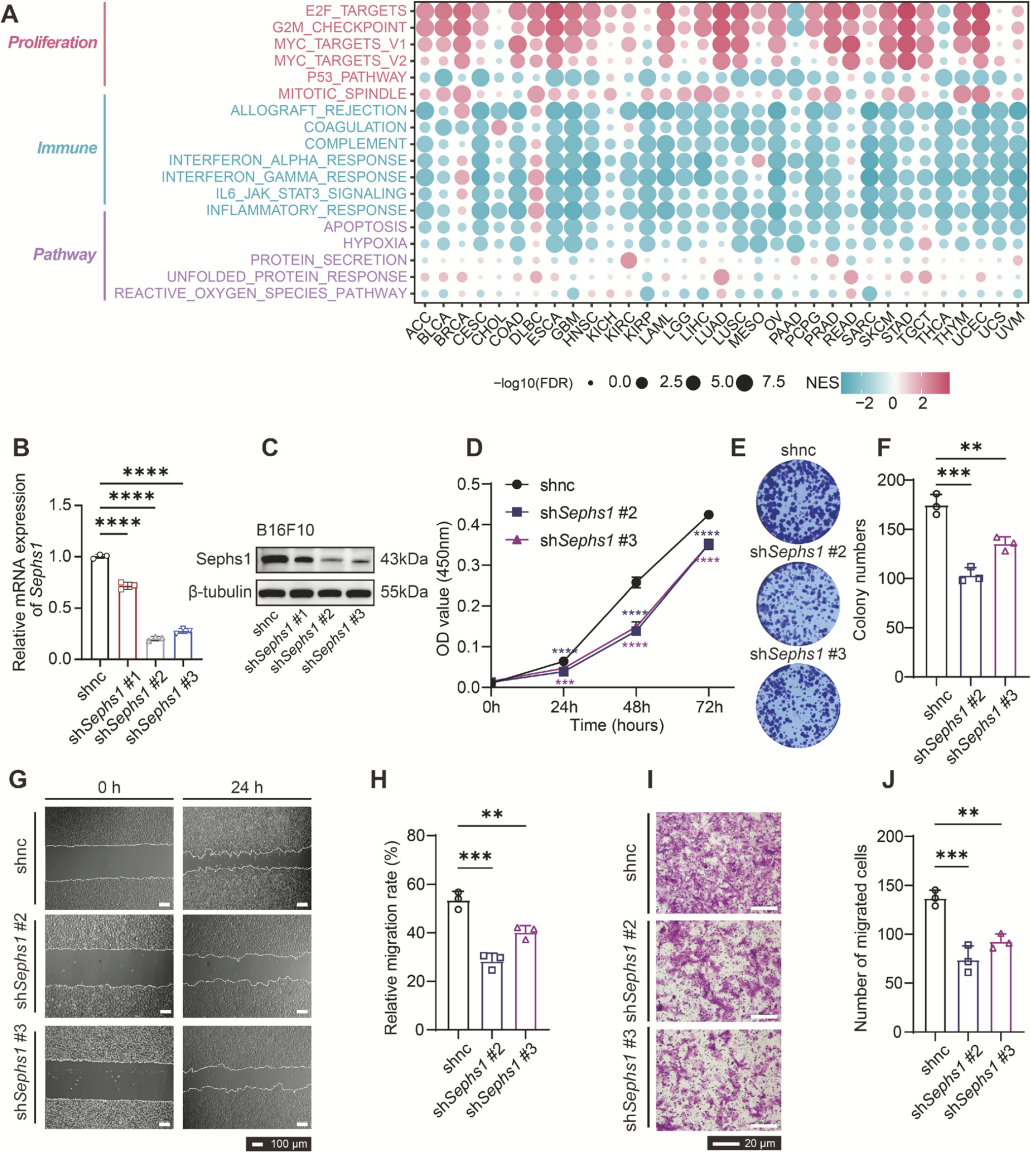
Effects of *Sephs1* knockdown on malignant phenotypes of murine melanoma cells. **A** Bubble plot showing the correlation between SEPHS1 expression and HALLMARK pathway activity scores across multiple cancer types, as determined by GSEA. Color indicates the normalized enrichment score (NES), and dot size represents -log_10_(FDR). **B** Quantitative RT-PCR analysis confirming knockdown efficiency of Sephs1 mRNA in B16F10 cells, normalized to control group. **C** Western blot analysis of Sephs1 protein levels in B16F10 cells, with β-tubulin as the loading control. **D** Cell proliferation analysis using CCK-8 assay after *Sephs1* knockdown. **E** Representative images of colony formation assay in shnc and *Sephs1* knockdown cells. **F** Quantification of colony numbers in colony formation assay. **G** Wound healing assay showing representative migration images at 0 and 24 h after *Sephs1* knockdown. **H** Quantification of migration area based on three independent visual fields. **I** Representative images from Transwell invasion assay of B16F10 cells after *Sephs1* knockdown. **J** Quantification of invaded cells per field. ****p < 0.0001, ***p < 0.001, **p < 0.01, *p < 0.05, ns *p* ≥ 0.05

To investigate the relationship between *SEPHS1* expression and tumor cell phenotypes, we performed pathway enrichment analysis using HALLMARK gene sets. *SEPHS1* expression was positively correlated with multiple proliferation-related pathways in over 80% of cancer types. In particular, high *SEPHS1* expression was consistently associated with MYC signaling, G2M checkpoint, and E2F target pathways, all of which are critical for cell cycle regulation (Fig. 5A).

To validate the biological relevance of SEPHS1 in specific cancer types, we selected B16F10 and MB49 cell lines as models for SKCM and BLCA, respectively (Fig. 5B, C, Figure S6A-B). CCK-8 assays and colony formation experiments showed that knockdown of *Sephs1* significantly suppressed the proliferation of both B16F10 and MB49 cells (Fig. 5D–F, Figure S6C-E). In vivo, subcutaneous transplantation of MB49-sh*Sephs1* cells into C57BL/6 J mice demonstrated that *Sephs1* knockdown markedly reduced tumor growth, as indicated by slower tumor progression and smaller tumor volume compared to control (Figure S6F-H). Immunohistochemical staining further confirmed a reduced proportion of Ki67-positive cells in Sephs1-depleted tumors, indicating decreased proliferative activity (Figure S6I-J).

Wound healing assays showed that *Sephs1* knockdown modestly reduced the migratory capacity of both B16F10 and MB49 cells. However, the difference was not statistically significant in MB49 cells (Fig. 5G–H, Figure S6K-L). Similarly, Transwell invasion assays revealed a slight reduction in invasive capacity in both cell lines, with a more pronounced effect observed in B16F10 cells (Fig. 5I, J, Figure S6M-N). Collectively, these findings suggest that SEPHS1 contributes to tumor cell proliferation and partially supports migratory and invasive phenotypes, reinforcing its potential role in cancer progression.

### SEPHS1 is associated with an immunosuppressive tumor microenvironment

To investigate the role of SEPHS1 in tumor immunity, we analyzed its association with immune microenvironment features. Except for pancreatic ductal adenocarcinoma, *SEPHS1* expression was negatively correlated with Immune Score and ESTIMATE Score across the remaining 30 cancer types, and also showed significant negative correlations with Stromal Score in the majority of tumors (Figure S7A). Correlation analysis with immune checkpoint genes revealed context-specific patterns: negative associations were observed in testicular germ cell tumors, thyroid carcinoma, and sarcoma, while positive correlations appeared in hepatocellular carcinoma, breast carcinoma, and bladder cancer (Figure S7B). TIMER analysis showed that *SEPHS1* expression was positively associated with MDSCs infiltration across most cancers, suggesting a role in promoting immunosuppressive cell recruitment (Figure S7C).

Further analysis using the TIP database indicated that high *SEPHS1* expression was negatively correlated with multiple steps of the cancer-immunity cycle in tumors such as adrenocortical carcinoma, hepatocellular carcinoma, lung adenocarcinoma, melanoma, and endometrial carcinoma, whereas a partial positive correlation was seen in breast cancer (Figure S7D). In addition, *SEPHS1* expression showed strong positive correlations with tumor mutational burden in selected cancers, including adrenocortical carcinoma, lung adenocarcinoma, and melanoma (Figure S7E). These findings suggest that SEPHS1 may contribute to immune evasion by modulating immune cell infiltration, checkpoint expression, and antitumor immune activation.

### High *SEPHS1* expression is associated with improved immunotherapy response in bladder cancer

To explore the clinical relevance of SEPHS1 in immunotherapy, we analyzed cohorts with available treatment response data [31–34]. In a bladder cancer cohort, the proportion of patients achieving complete or partial response (CR/PR) was significantly higher in the *SEPHS1* high-expression group. In contrast, no apparent association was observed between *SEPHS1* expression and immunotherapy response in melanoma cohorts (Figure S8A).

In the Mariathasan et al. bladder cancer cohort, *SEPHS1* high expression was associated with higher tumor mutational burden (Figure S8B) and distinct mutation patterns, including enriched TP53 and RB1 mutations, while FGFR3 and STAG2 mutations were more common in the low-expression group (Figure S8D). *SEPHS1* high expression was also linked to elevated neoantigen burden (Figure S8C) and upregulation of multiple DNA damage repair-related pathways, such as homologous recombination, mismatch repair, and cell cycle (Figure S8E).

These findings suggest that *SEPHS1* high expression may indicate enhanced genomic instability and immunogenicity, supporting its potential as a biomarker for immunotherapy response in bladder cancer.

### *Sephs1* knockdown promotes CD8⁺ T cell infiltration and enhances effector function in vivo

**Fig. 6:**
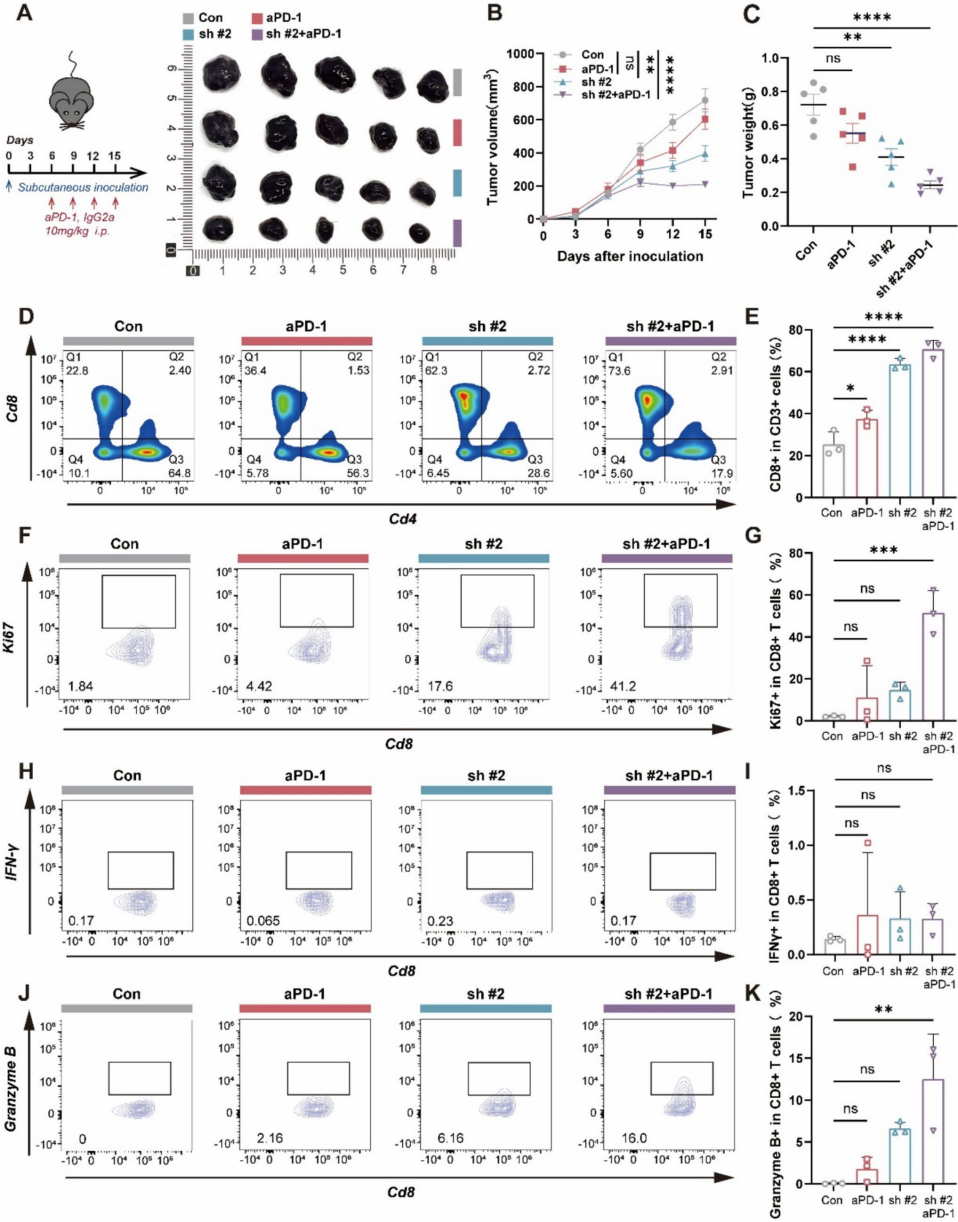
*Sephs1* knockdown enhances Anti-PD-1 efficacy by promoting CD8⁺ T cell infiltration and effector function in vivo. **A** Experimental schematic and representative tumor images from four groups: control (shnc + IgG2a), anti-PD-1 treatment (shnc + Anti-PD-1), *Sephs1* knockdown (sh*Sephs1* #2 + IgG2a), and combination therapy (sh*Sephs1* #2 + Anti-PD-1). **B** Tumor growth curves showing tumor volume over time (days after inoculation). Statistical comparisons were performed based on tumor volume at day 15 post-inoculation. **C** Tumor weight.All treatment groups were compared against the control group (Con). **D**, **E** Flow cytometry density plots and quantification of CD4⁺ and CD8⁺ T cell infiltration in tumors. **F**, **G** Density plots and quantification of Ki-67 + proliferating CD8⁺ T cells. (H, I) Density plots and quantification of IFN-γ expression in CD8⁺ T cells. (J, K) Density plots and quantification of granzyme B expression in CD8⁺ T cells. ****p < 0.0001, ***p < 0.001, **p < 0.01, *p < 0.05, ns *p* ≥ 0.05

To evaluate the in vivo effects of *Sephs1* knockdown on tumor growth and immunotherapy response, we established a subcutaneous B16F10 melanoma model in C57BL/6 J mice, followed by anti-PD-1 treatment. Compared to controls, *Sephs1* knockdown significantly reduced tumor volume and weight by day 15 post-implantation. The combination of *Sephs1* knockdown and anti-PD-1 therapy resulted in further tumor suppression (Fig. 6A–C).

Flow cytometry revealed an evident increase increase in CD8⁺ T cell infiltration and a concurrent reduction in CD4⁺ T cells in the *Sephs1* knockdown group, suggesting a shift toward cytotoxic T cell-dominated immune responses (Fig. 6D, E). Although the proportion of IFN-γ^+^CD8⁺ T cells remained unchanged, Granzyme B^+^ CD8⁺ T cells were markedly elevated following combination therapyCD8⁺(*p* < 0.001), indicating enhanced effector function (Fig. 6F–K).

These findings suggest that *Sephs1* knockdown promotes CD8⁺ T cell infiltration and activation in the tumor microenvironment, thereby enhancing the antitumor efficacy of PD-1 blockade.

### SEPHS1 suppression enhances T cell mediated antitumor activity in melanoma

**Fig. 7:**
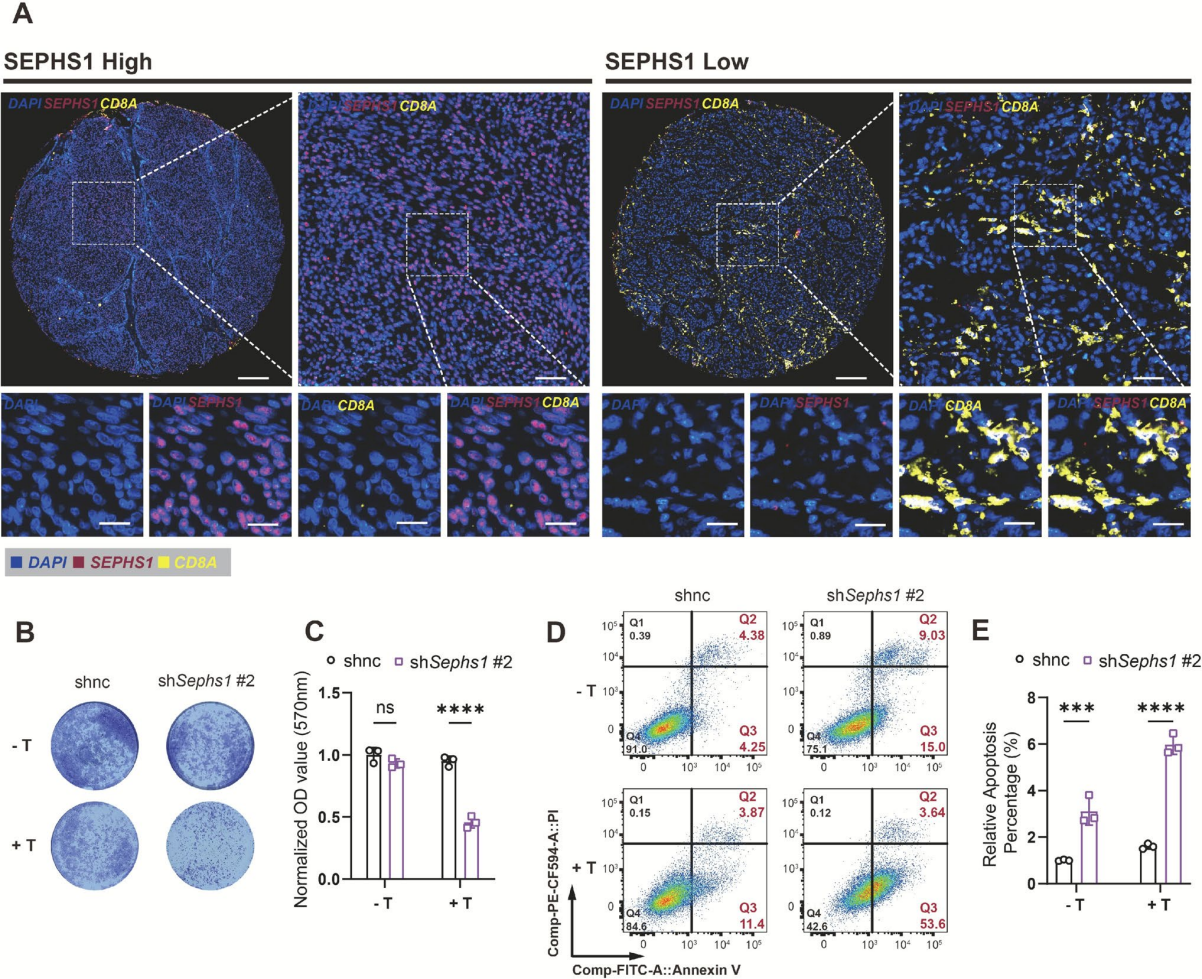
*Sephs1* knockdown enhances T cell mediated tumor cell apoptosis and reduces tumor cell survival. **A** Representative multiplex immunofluorescence images showing the spatial distribution of SEPHS1 (red) and CD8A.^+^ T cells (yellow) in melanoma tissue. Nuclei are stained with DAPI (blue). Images include whole-tissue sections and high-magnification views. Scale bars: 200 μm (whole view), 50 μm (zoomed view). **B** Crystal violet staining of tumor cells after co-culture with or without T cells, comparing *Sephs1* knockdown and control groups. **C** Quantification of crystal violet staining by OD value showing reduced tumor cell density in the *Sephs1* knockdown group after T cell co-culture. **D** Flow cytometry dot plots of Annexin V-FITC/PI staining showing apoptosis of tumor cells following co-culture with T cells. **E** Quantification of apoptotic cells showing significantly increased apoptosis in *Sephs1* knockdown cells, further enhanced by T cell co-culture. ****p < 0.0001, ***p < 0.001, **p < 0.01, *p < 0.05, ns *p* ≥ 0.05

Multiplex immunofluorescence analysis of human melanoma tissues revealed that SEPHS1 was primarily localized in tumor cell cytoplasm and absent in stromal or immune cells. Regions with high *SEPHS1* expression showed reduced CD8⁺ T cell infiltration, forming an “immune-excluded” phenotype, whereas low *SEPHS1* expression was associated with increased CD8⁺ T cell infiltration and direct tumor contact (Fig. 7A).

To evaluate the functional impact of SEPHS1 suppression, a direct co-culture system was established. In the absence of T cells, *Sephs1* knockdown led to a mild reduction in tumor cell viability. Upon T cell co-culture, cell viability further decreased in the knockdown group compared with controls, indicating enhanced T cell mediated cytotoxicity (Fig. 7B, C). Flow cytometry confirmed a marked increase in apoptosis following *Sephs1* knockdown, which further intensified upon T cell co-culture (Fig. 7D, E).

These findings demonstrate that SEPHS1 inhibition suppresses tumor growth and enhances CD8⁺ T cell cytotoxicity, supporting its potential as a therapeutic target to improve immunotherapy outcomes in melanoma.

### SEPHS1 regulates immune evasion in melanoma by suppressing chemokine signaling and antigen presentation

**Fig. 8:**
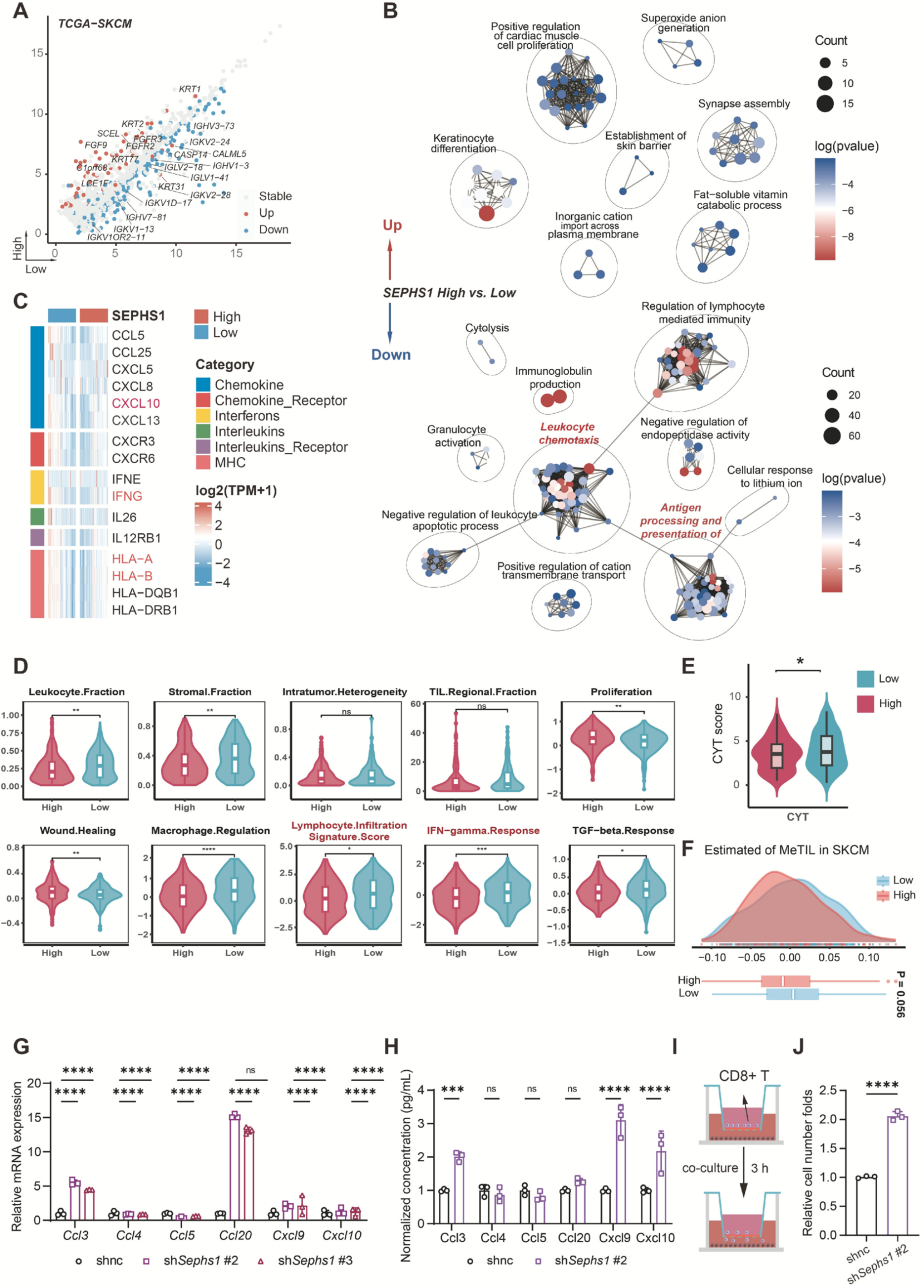
SEPHS1 regulates immune signaling and impairs CD8⁺ T cell recruitment in melanoma. **A** Angled volcano plot showing differentially expressed genes between SEPHS1 high and low expression groups in the TCGA-SKCM cohort. Red indicates genes upregulated in the high SEPHS1 group, blue indicates genes upregulated in the low SEPHS1 group. **B** GO enrichment network analysis of differentially expressed genes between SEPHS1 high and low groups. Node size represents enrichment strength, and edges represent functional similarity. **C** Heatmap showing differential expression of immune-regulatory molecules, including chemokines, interferons, interleukins, and MHC-related genes between SEPHS1 high and low groups. **D** Violin plots showing differences in immune-related signature scores between SEPHS1 high and low groups. Wilcoxon rank-sum test was used for statistical analysis. **E** CYT scores between SEPHS1 high and low expression groups. **F** Distribution of MeTIL scores between SEPHS1 high and low groups, calculated by PCA-based immune deconvolution and compared using the Wilcoxon test. **G** RT-qPCR analysis of T cell-related chemokine expression in B16F10 cells after *Sephs1* knockdown. **H** ELISA quantification of chemokine secretion levels after *Sephs1* knockdown. **I** Schematic of Transwell migration assay showing CD8⁺ T cells in the upper chamber and B16F10 tumor cells in the lower chamber. **J** Quantification of migrated CD8⁺ T cells shown as fold change relative to control.****p < 0.0001, ***p < 0.001, **p < 0.01, *p < 0.05, ns *p* ≥ 0.05

To investigate how SEPHS1 shapes the immunosuppressive landscape in melanoma, we analyzed transcriptomic profiles from the TCGA-SKCM cohort. Tumors with high *SEPHS1* expression showed increased levels of epidermal differentiation genes including *KRT1*, *KRT2*, and *SCEL*, and sciellin, suggesting a terminal differentiation phenotype that may limit neoantigen presentation. At the same time, immunoglobulin genes such as *IGKV2-28*, *IGKV1-13*, and *IGHV3-73* were downregulated, indicating reduced humoral immune activity (Fig. 8A).

Gene Ontology enrichment revealed that immune-related processes, including leukocyte chemotaxis, immunoglobulin production, and antigen processing, were significantly suppressed in the *SEPHS1* high-expression group (Fig. 8B). In contrast, tumors with low *SEPHS1* expression displayed higher expression of interferon-gamma, *CXCL10*, and chemokine receptors such as *CXCR3* and *CXCR6*, along with elevated *HLA-A* and *HLA-B* levels, suggesting enhanced antigen presentation and T cell recruitment (Fig. 8C).

Immune infiltration analysis further showed that the *SEPHS1* low-expression group had higher lymphocyte infiltration, stronger interferon responses, increased cytolytic activity, and higher MeTIL scores, reflecting more active T cell and NK cell responses (Fig. 8D–F).

To validate these findings, we examined chemokine regulation in B16F10 melanoma cells. Knockdown of *Sephs1* increased mRNA and protein levels of CCL3, CXCL9, and CXCL10, supporting the role of SEPHS1 in restraining chemokine production (Fig. 8G–H). Finally, Transwell migration assays demonstrated that *Sephs1* knockdown enhanced CD8 positive T cell migration by more than two-fold compared to control cells, confirming its functional impact on T cell recruitment (Fig. 8I, J).

Overall, these results indicate that SEPHS1 promotes immune evasion by limiting chemokine signaling and antigen presentation, and its inhibition can enhance antitumor immune responses in melanoma.

## Discussion

This study systematically characterized the expression heterogeneity and epigenetic regulation of selenium metabolism-related genes across cancers, with a particular focus on SEPHS1. Multi-omics analyses revealed consistently elevated expression of *AHCY*, *BUD23*, *LCMT1*, *MARS1*, *METTL6*, *SCLY* and *SEPHS1* in various tumor types. Previous studies reported *SEPHS1* overexpression in gastrointestinal malignancies such as rectal and gastric adenocarcinomas [44, 45]. A recent pan-cancer study further demonstrated that SEPHS1 expression was broadly associated with tumor immunity and patient prognosis, showing favorable outcomes in low-grade glioma but poorer survival in hepatocellular carcinoma [46]. While that study primarily established correlative evidence, our work extends these findings by functionally linking SEPHS1 to immune infiltration and therapeutic responsiveness. Specifically, we demonstrate that SEPHS1 shapes the tumor immune landscape and modulates the efficacy of anti-PD-1 therapy, thereby providing mechanistic insight into its prognostic relevance and tumor-type specificity. These contrasting patterns imply functional divergence dependent on tumor context and associated signaling pathways.

Additional selenium metabolism-related genes also showed oncogenic roles; for instance, AHCY modulates epigenetic regulation via the methionine cycle and promotes MYC-driven proliferation in colorectal cancer [47], while BUD23 shapes the tumor microenvironment in hepatocellular carcinoma by impairing T cell and macrophage responses and downregulating immune checkpoint gene expression [48]. To further explore the immunological relevance of selenium metabolism, we established the SAS across cancers. SAS showed negative correlations with both IFN-γ and TGF-β response pathways, and was inversely associated with most T cell subsets, including CD8⁺ T cells and activated memory CD4⁺ T cells, suggesting a potential immunosuppressive role of enhanced selenium metabolism. Interestingly, we observed a positive correlation between SAS and resting memory CD4⁺ T cells in several tumor types. This pattern may indicate a shift toward an immune state dominated by quiescent or long-lived T cells with limited effector activation, reflecting immune adaptation rather than active cytotoxic engagement. Transcriptomic datasets associated *HS1* expression with activation of MYC signaling and cell cycle progression, and functional assays further confirmed its role in promoting malignant behavior in melanoma. Silencing of *Sephs1* reduced tumor cell proliferation, migration and invasion, consistent with reports that identified SEPHS1 as a driver of metastasis in hepatocellular carcinoma via the TGF-β/SMAD pathway [49]. Furthermore, SEPHS1 has been included in a nine-gene risk model based on amino acid metabolism in hepatocellular carcinoma. This risk model independently predicts poor clinical outcomes, with high-risk patients demonstrating significantly shorter overall survival [50]. These findings underscore the oncogenic potential of SEPHS1 in specific cancer settings.

Beyond tumor biology, *SEPHS1* encodes a key rate-limiting enzyme in selenium metabolism and plays an essential role in maintaining redox balance. It catalyzes the production of selenophosphate, providing selenium donors for selenoproteins such as glutathione peroxidases (GPX) and thioredoxin reductases (TXNRD) [19]. Under physiological conditions, SEPHS1 is indispensable for embryonic development, neuronal activity and skeletal system integrity [40, 51]. Functional loss of SEPHS1 results in excessive reactive oxygen species, DNA damage and cell cycle arrest [52]. These consequences are implicated in multiple diseases [53]. For example, in osteoarthritis, downregulation of SEPHS1 impairs selenoprotein synthesis in chondrocytes, leading to reduced antioxidant capacity and cartilage degeneration [19]. In vascular disease, deficiency of SEPHS1 hampers endothelial migration and angiogenesis, while promoting DNA damage [54]. Together, these studies illustrate the systemic importance of SEPHS1 in oxidative stress regulation.

Integrated CRISPR screening and immunotherapy datasets uncovered a novel role of SEPHS1 in immune regulation. In melanoma, silencing *Sephs1* enhanced CD8⁺ T cell infiltration and improved the efficacy of anti-PD-1 therapy. This observation was supported by pan-cancer transcriptomic analyses, where *SEPHS1* expression was inversely correlated with gene expression signatures associated with immune activation. Immunofluorescence analysis revealed exclusion of CD8⁺ T cells in tumors with high *SEPHS1* expression, whereas knockdown of *Sephs1* led to increased expression of CXCL9 and CXCL10, enhanced T cell migration, and improved effector function. Interestingly, SEPHS1 appears to exert distinct immune effects in different cancer types. In melanoma, SEPHS1 promotes immune exclusion and correlates with MDSC infiltration. In contrast, in bladder cancer, high *SEPHS1* expression is associated with increased tumor mutational burden, elevated PD-L1 expression, and improved immunotherapy response, possibly due to enhanced neoantigen release. These findings suggest that SEPHS1 may modulate tumor immunity through either immunosuppressive or immunogenic pathways depending on immune context, TP53 status and tumor type. This tumor-type specificity may reflect intrinsic differences in immune microenvironment composition and baseline immunogenicity between melanoma and bladder cancer. The limited sample size of melanoma immunotherapy cohorts may also contribute to the lack of statistical significance despite a consistent biological trend.

From a translational perspective, SEPHS1 may function as a context-dependent metabolic regulator that links selenium metabolism to tumor immunity. In melanoma, its inhibition was associated with enhanced immune infiltration and improved response to PD-1 blockade in preclinical models, whereas in bladder cancer, high SEPHS1 expression may be associated with a more immunogenic phenotype and improved response to checkpoint blockade. These observations suggest that SEPHS1 may serve as both a potential biomarker and therapeutic target, with its clinical implications depending on tumor immune context and genomic background.

From a metabolic and nutritional standpoint, SEPHS1 may represents a key intersection between selenium metabolism, redox homeostasis, and immune regulation. Given that selenium is an essential micronutrient with established clinical use, modulation of selenium metabolism may offer a potential avenue for therapeutic intervention, though further validation is required to assess feasibility and safety. Such integration of nutrient metabolism and immunotherapy provides a conceptual framework for future exploration of “precision nutrimetabolic immunotherapy” [55].

Despite these findings, several knowledge gaps remain. SEPHS1 and SEPHS2 perform distinct biochemical functions. While SEPHS2 catalyzes de novo selenophosphate synthesis from inorganic selenium, SEPHS1 primarily recycles selenium from L-selenocysteine [56]. It is plausible that high intracellular L-selenocysteine concentrations may inhibit SEPHS1 expression or activity via feedback regulation, thus preventing excessive selenium accumulation and redox imbalance. Testing this hypothesis will require selenium titration experiments, metabolomics, and conditional knockout models. Additionally, the precise mechanism by which SEPHS1 regulates immune signaling remains unclear. Although SEPHS1 influences interferon signaling and the CXCL9/CXCL10 axis, it is uncertain whether this occurs via transcriptional regulation or through modulation of redox-sensitive pathways such as NF-κB or STAT1. Cross-talk with ER stress, metabolic rewiring or DNA repair should also be explored. Notably, current in vivo validation is limited to melanoma models. The role of SEPHS1 may vary across tumors with different immunogenicity. In immune-inflamed tumors, SEPHS1 inhibition may increase immunogenicity through genomic instability but carry toxicity risks. In immune-cold tumors, SEPHS1 may regulate immune evasion via metabolic remodeling. Therefore, evaluating SEPHS1 function across tumor immune subtypes and identifying predictive biomarkers such as PD-L1, TMB, or selenium status will be crucial. Future studies should aim to establish SEPHS1-centered regulatory networks using multi-omics approaches and expand validation across various tumor types and immune contexts. Investigating dietary selenium modulation and targeted delivery strategies may further enhance translational potential and overcome immunotherapy resistance.

## Conclusions

This study identifies SEPHS1 as a critical immunometabolic regulator that contributes to immune evasion in melanoma. SEPHS1 limits CD8⁺ T cell infiltration and effector activation by repressing chemokine expression, thereby weakening antitumor immunity. Genetic silencing of SEPHS1 not only impairs tumor cell growth but also promotes T cell infiltration and effector function, ultimately enhancing the efficacy of anti–PD-1 therapy in vivo. These findings suggest that targeting SEPHS1 may represent a promising strategy to overcome immune resistance and improve immunotherapy response in melanoma.

## Supplementary Information

Below is the link to the electronic supplementary material.

Supplementary file1 (DOCX 27789 kb)

Supplementary file2 (XLSX 36 kb)

## Data Availability

All data analyzed in this study were obtained from publicly available databases. Bulk RNA sequencing data, corresponding clinical annotations, somatic mutation, CNV, and DNA methylation profiles were retrieved from the UCSC Xena platform, based on TCGA and GTEx datasets. Proteomic data were obtained from the CPTAC. Single-cell RNA sequencing datasets, including GSE210347 and GSE215120, were downloaded from the GEO database. Melanoma immunotherapy cohorts GSE78220 (Hugo et al.) and GSE91061 (Riaz et al.) were also acquired from GEO, while PRJEB23709 (Gide et al.) was obtained from the TIGER database The urothelial carcinoma cohort IMvigor210 (Mariathasan et al.) was accessed through the IMvigor210CoreBiologies R package, and GSE176307 (Rose et al.) was downloaded from GEO. Gene sets for selenium metabolism were retrieved from the MsigDB, Single-cell expression visualization and annotation were performed using the TISCH.

## Abbreviations

CCLE: Cancer cell line encyclopedia
CNV: Copy number variation
CPTAC: Clinical proteomic tumor analysis consortium
CR: Complete response
CTLA-4: Cytotoxic T-lymphocyte-associated protein 4
CYT: Cytolytic activity
DFI: Disease-free interval
DSS: Disease-specific survival
ELISA: Enzyme linked immunosorbent assay
ERAP1: Endoplasmic reticulum aminopeptidase 1
GEO: Gene expression omnibus
GTEx: Genotype-tissue expression
GZMA: Granzyme A
GZMB: Granzyme B
ICIs: Immune checkpoint inhibitors
IFN-γ: Interferon-gamma
IHC: Immunohistochemistry
ISGs: Interferon-stimulated genes
JAK1: Janus kinase 1
MDSCs: Myeloid-derived suppressor cells
MFI: Mean fluorescence intensity
MHC-I: Major histocompatibility complex class I
mIHC: Multiplex immunohistochemical
MM: Multiple myeloma
NALs: Neoantigen loads
OS: Overall survival
PCA: Principal component analysis
PD: Progressive disease
PD-1: Programmed cell death protein 1
PD-L1: Programmed death-ligand 1
PFI: Progression-free interval
PR: Partial response
PRF1: Perforin
SCNAs: Somatic copy number alterations
SD: Stable disease
Sec: Selenocysteine
SeP: Selenophosphate
SEPHS1: Selenophosphate synthetase 1
SEPHS2: Selenophosphate synthetase 2
SNVs: Single nucleotide variants
SOCS1: Suppressor of cytokine signaling 1
STAT1: Signal transducer and activator of transcription 1
TAP1: Transporter associated with antigen processing 1
TAP2: Transporter associated with antigen processing 2
TCGA: The cancer genome atlas
TISCH: Tumor immune single-cell hub
TMB: Tumor mutation burden
TME: Tumor microenvironment
TRAF3: TNF receptor-associated factor 3
UMAP: Uniform manifold approximation and projection
WB: Western blot
