# Supplementary material for: Dissecting the role of SEPHS1 in shaping an immunosuppressive microenvironment to promote tumor progression
Source: Cancer Immunol Immunother. 2025 Dec 24;75(1):29. doi: 10.1007/s00262-025-04253-3 (PMC12738508; doi:10.1007/s00262-025-04253-3)
Supplement: Supplementary file 1 — Supplementary file1 (DOCX 27789 kb) [file 262_2025_4253_MOESM1_ESM.docx]

**Supplementary Figures**

**Dissecting the Role of SEPHS1 in Shaping an Immunosuppressive Microenvironment to Promote Melanoma Progression**

**
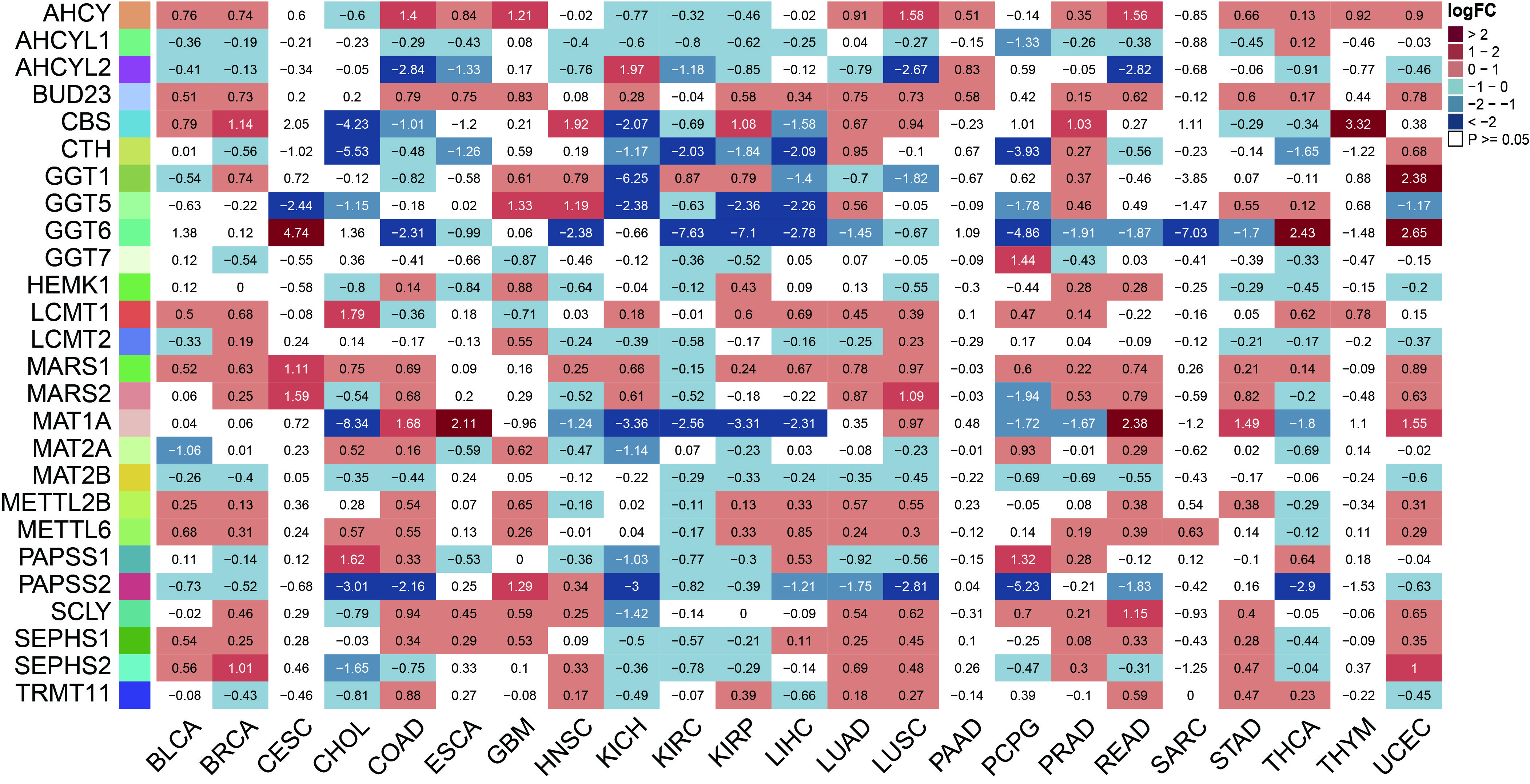
**

**Figure S1. Differential expression and methylation regulation of selenium metabolism related genes across cancers.** Heatmap showing the expression differences of selected genes between tumor and normal tissues across various cancer types. Red indicates higher expression in tumors, blue indicates lower expression, and color intensity reflects the magnitude of difference. White indicates no statistically significant difference (*p* ≥ 0.05).


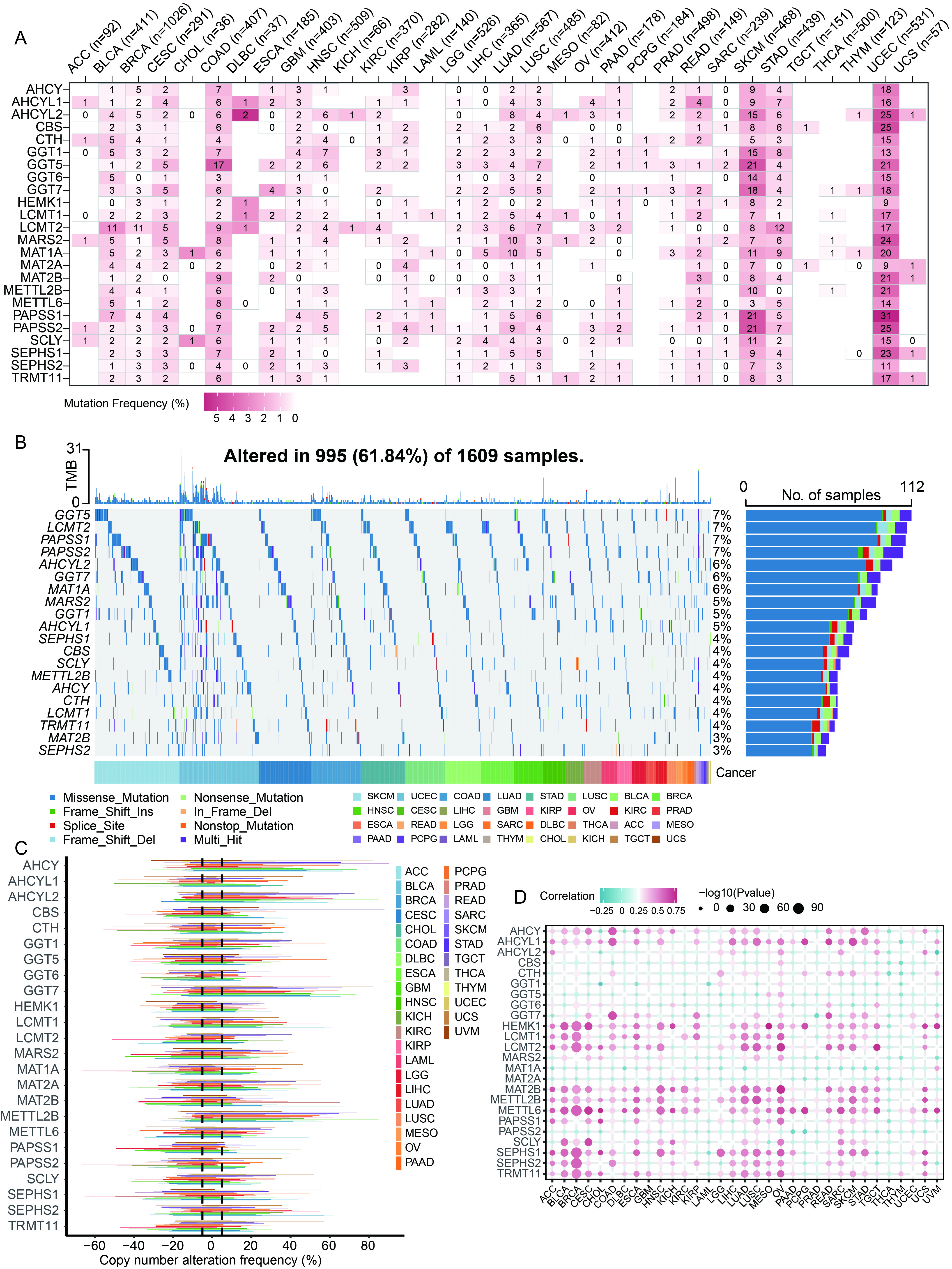


**Figure S2. Genomic alteration landscape of selenium metabolism related genes across cancers.** (A) Heatmap showing the distribution of single nucleotide variant (SNV) mutation frequencies of selenium metabolism related genes across different cancer types. (B) Waterfall plot illustrating the mutation types and frequencies of selenium metabolism related genes in various cancers. (C) Bar chart showing the distribution of somatic copy number alterations (SCNAs) of selenium metabolism–related genes across multiple cancer types. The x-axis represents copy number alteration frequency (%), with positive values indicating amplification and negative values indicating deletion..(D) Spearman correlation analysis between SCNA levels and mRNA expression of selenium metabolism-related genes across cancers.


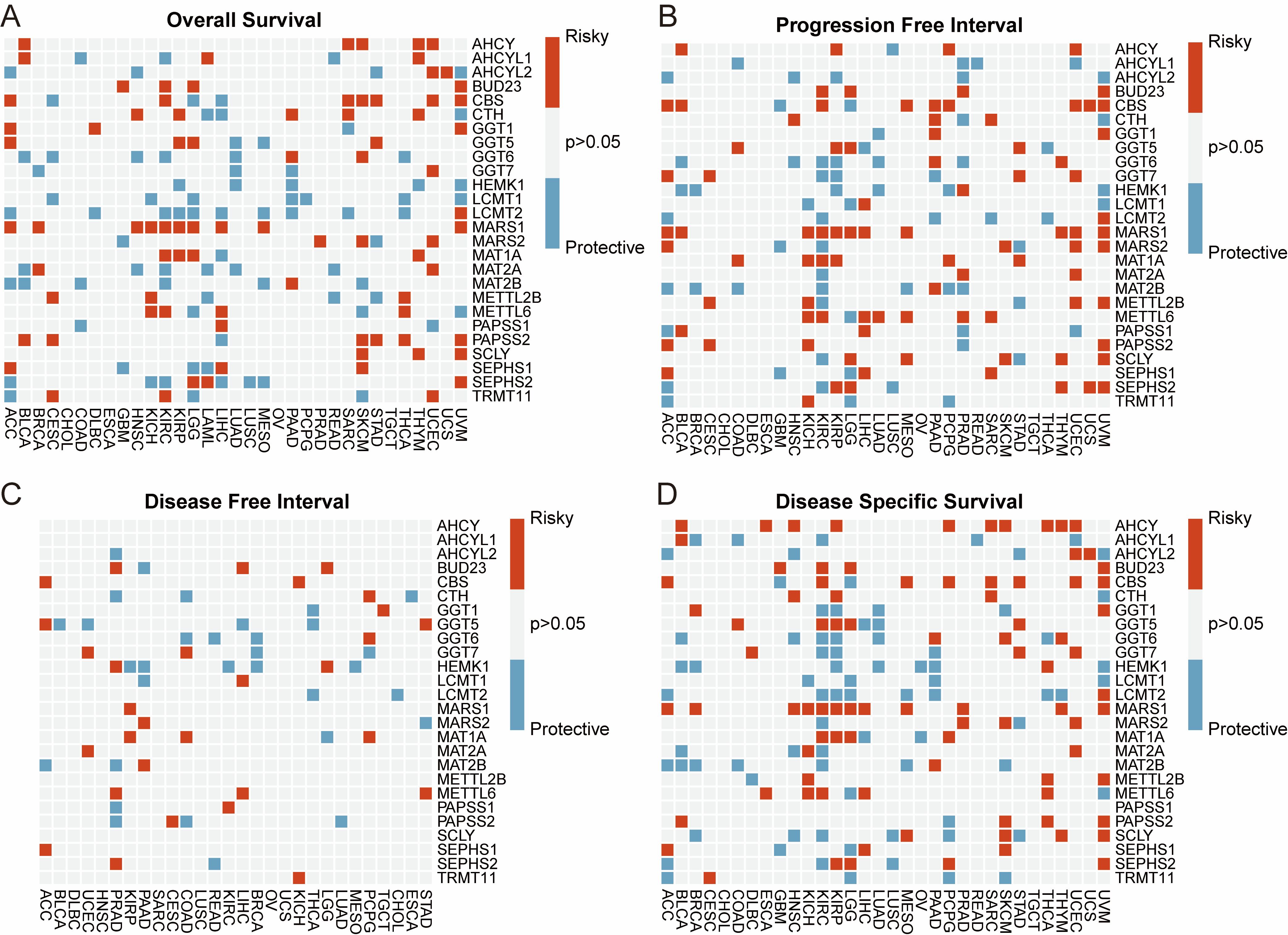


**Figure S3. Prognostic significance of selenium metabolism related genes across pan-cancer.** (A-D) Univariate Cox regression analysis was performed to evaluate the association between selenium metabolism related gene expression and four clinical survival endpoints: (A) Overall Survival (OS), (B) Progression-Free Interval (PFI), (C) Disease-Free Interval (DFI), and (D) Disease-Specific Survival (DSS). Each square represents the prognostic role of a given gene in a specific cancer type. Red squares indicate risk factors (hazard ratio > 1), blue squares indicate protective factors (hazard ratio < 1), and gray squares denote non-significant associations (*p* > 0.05)

**
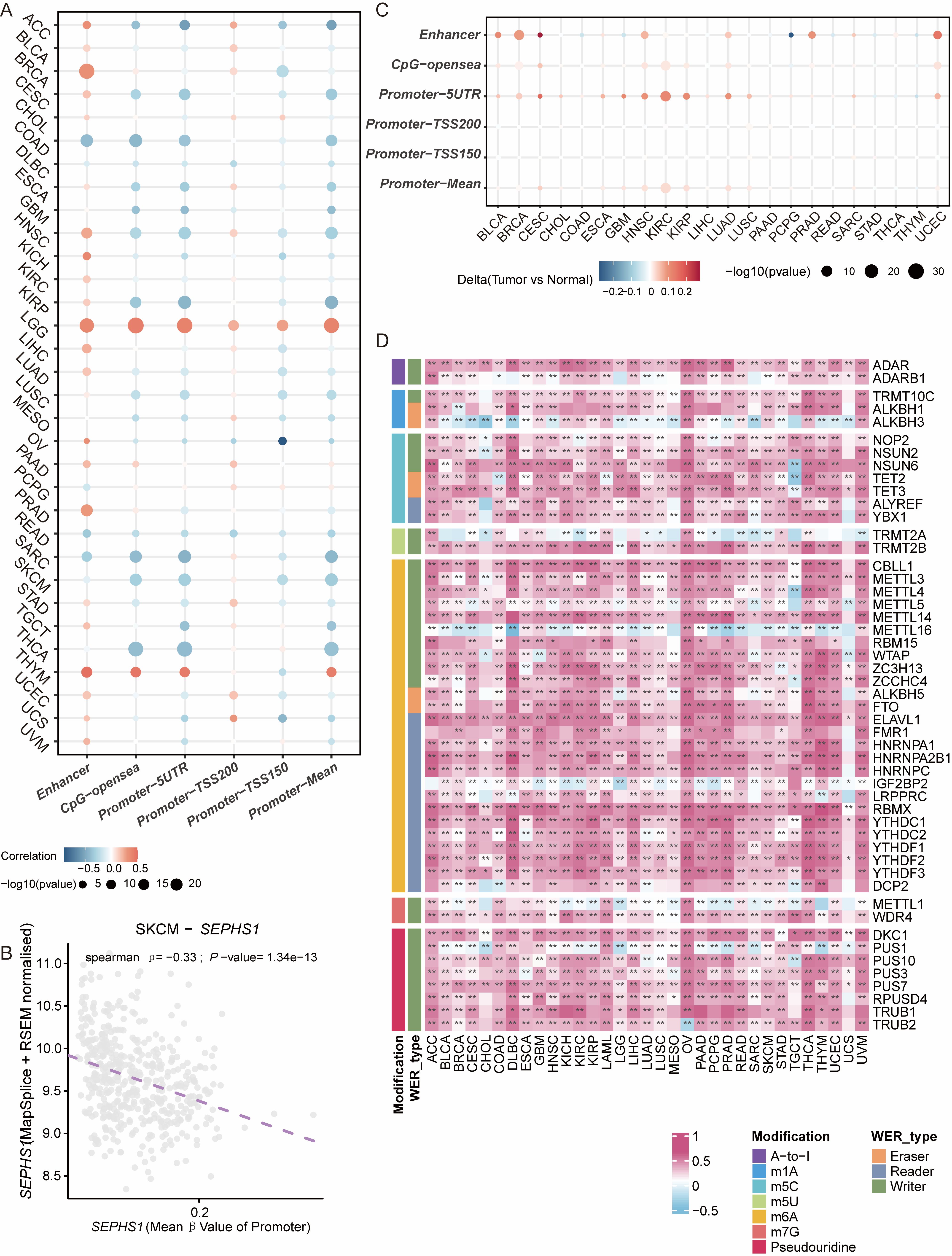
Figure S4. Correlation between SEPHS1 mRNA expression and epigenetic regulation across cancers.** (A) Spearman correlation between SEPHS1 mRNA expression and DNA methylation levels across enhancer and promoter regions (including 5′UTR, TSS200, TSS1500, and overall promoter average) in 33 cancer types. Dot color indicates the direction of correlation, and size reflects statistical significance. (B) A representative scatter plot showing the negative correlation between SEPHS1 promoter methylation (mean β-value) and mRNA expression in SKCM (skin cutaneous melanoma).

(C) Differences in SEPHS1 DNA methylation levels between tumor and normal tissues across various genomic regions in 22 cancer types. Dot color represents the methylation difference (tumor vs. normal), and size indicates -log10(p-value). (D) Heatmap displaying Spearman correlation between SEPHS1 mRNA expression and RNA modification regulators (writers, erasers, and readers) across five major RNA modification types (A-to-I, m^1^A, m^5^C, m^6^A, m^7^G, pseudouridine). **p* < 0.05, ***p* < 0.01.

**
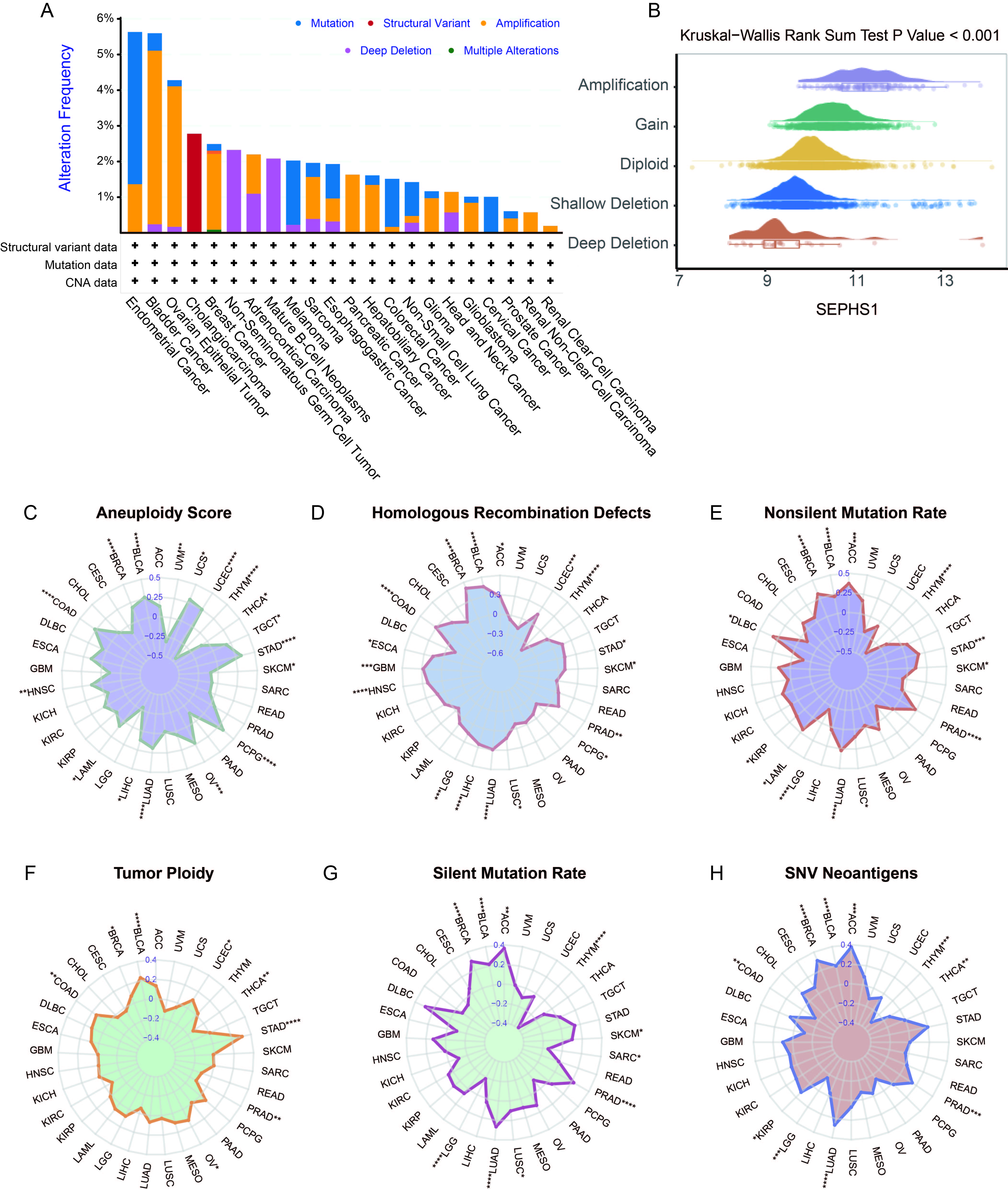
Figure S5. Genomic alterations and instability associated with SEPHS1 expression across cancer types.** (A) Distribution of SEPHS1 genomic alterations, including amplification, shallow deletion, deep deletion, mutation, and structural variants across different cancer types. (B) SEPHS1 mRNA expression levels under different CNV states (amplification, gain, diploid, shallow deletion, and deep deletion). Statistical differences were assessed using the Kruskal-Wallis rank-sum test. (C-H) Correlation between SEPHS1 expression and genomic instability indicators across cancers, including: (C) Aneuploidy score, (D) Homologous recombination defects, (E) Nonsilent mutation rate, (F) Tumor ploidy, (G) Silent mutation rate, and (H) SNV neoantigen load. *****p* < 0.0001, ****p* < 0.001, ***p* < 0.01, **p* < 0.05.


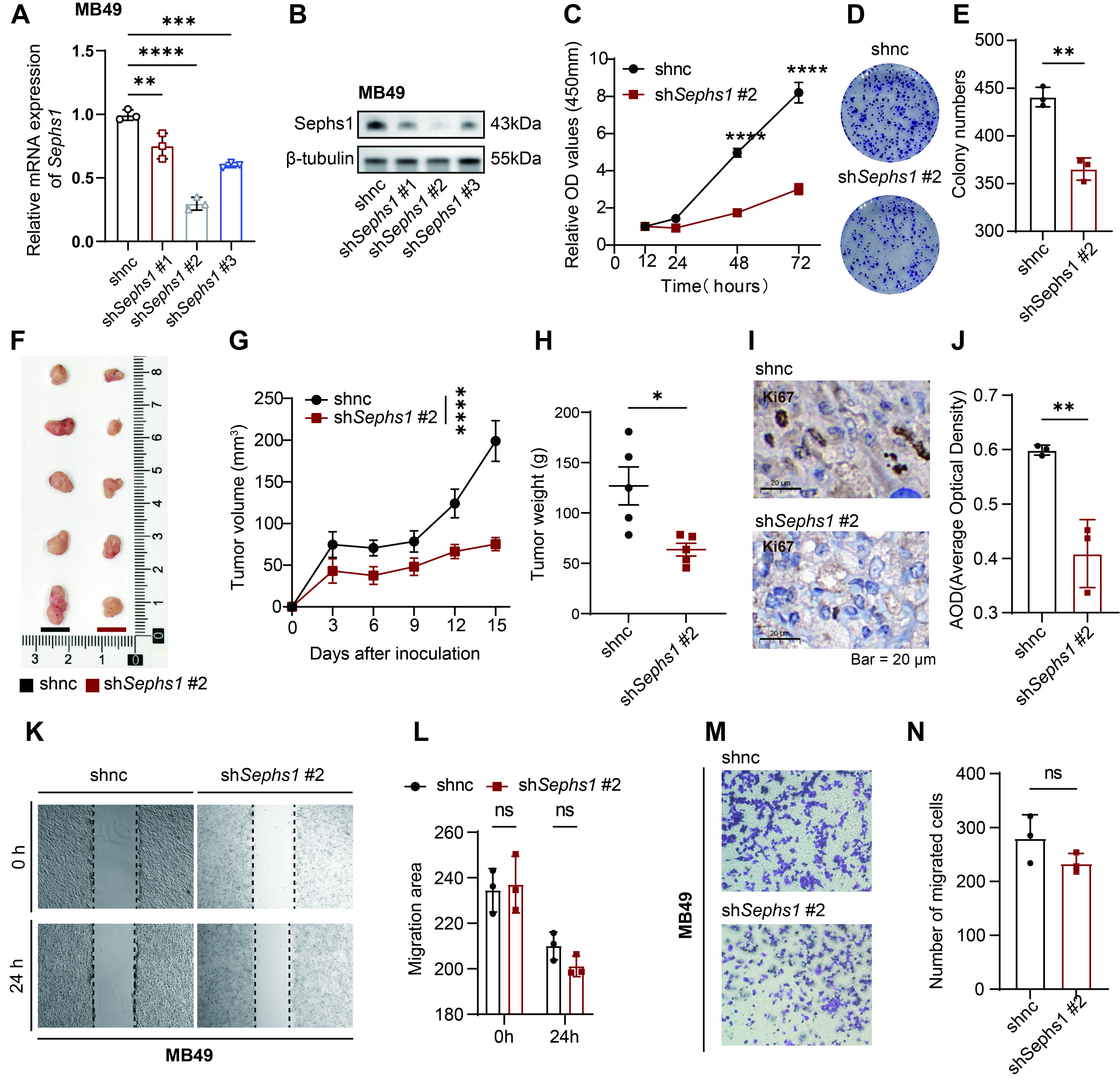
**Figure S6. SEPHS1 is essential for bladder cancer cell proliferation and tumor progression.** (A) RT-qPCR showing the mRNA knockdown efficiency of Sephs1 in MB49 cells (mean ± SD, n = 3). (B) Western blot confirming Sephs1 protein knockdown in MB49 cells (β-tubulin as loading control). (C-E) Effects of Sephs1 knockdown on MB49 cell proliferation. (C) Cell viability was measured by CCK-8 assay at 0, 12, 24, 48, and 72 h. (D) Representative images of colony formation; (E) quantification of colony numbers (mean ± SD, n = 3). (F-H) In vivo tumor growth analysis in C57BL/6 mice subcutaneously injected with Sephs1-knockdown or control MB49 cells (1 × 10⁶ cells per mouse). (F) Representative tumor images; (G) Tumor volume growth curves. Asterisks indicate statistical significance between groups at the final measurement time point (day 15); (H) tumor weight at endpoint (mean ± SD, n = 5-6 per group). (I-J) Immunohistochemical staining for Ki-67 in tumor sections; (I) representative IHC images; (J) quantification of average optical density (AOD) of Ki-67-positive areas. (K-L) Wound-healing assays assessing migration ability of MB49 cells after Sephs1 knockdown; (K) representative images at 0 and 24 h; (L) quantification of migration area (mean ± SD, n = 3). (M-N) Transwell invasion assays of Sephs1-knockdown MB49 cells; (M) representative invaded cell images; (N) quantification of invaded cells per field (mean ± SD, n = 3). **p* < 0.05, ***p* < 0.01, ****p* < 0.001, *****p* < 0.0001, ns = not significant.


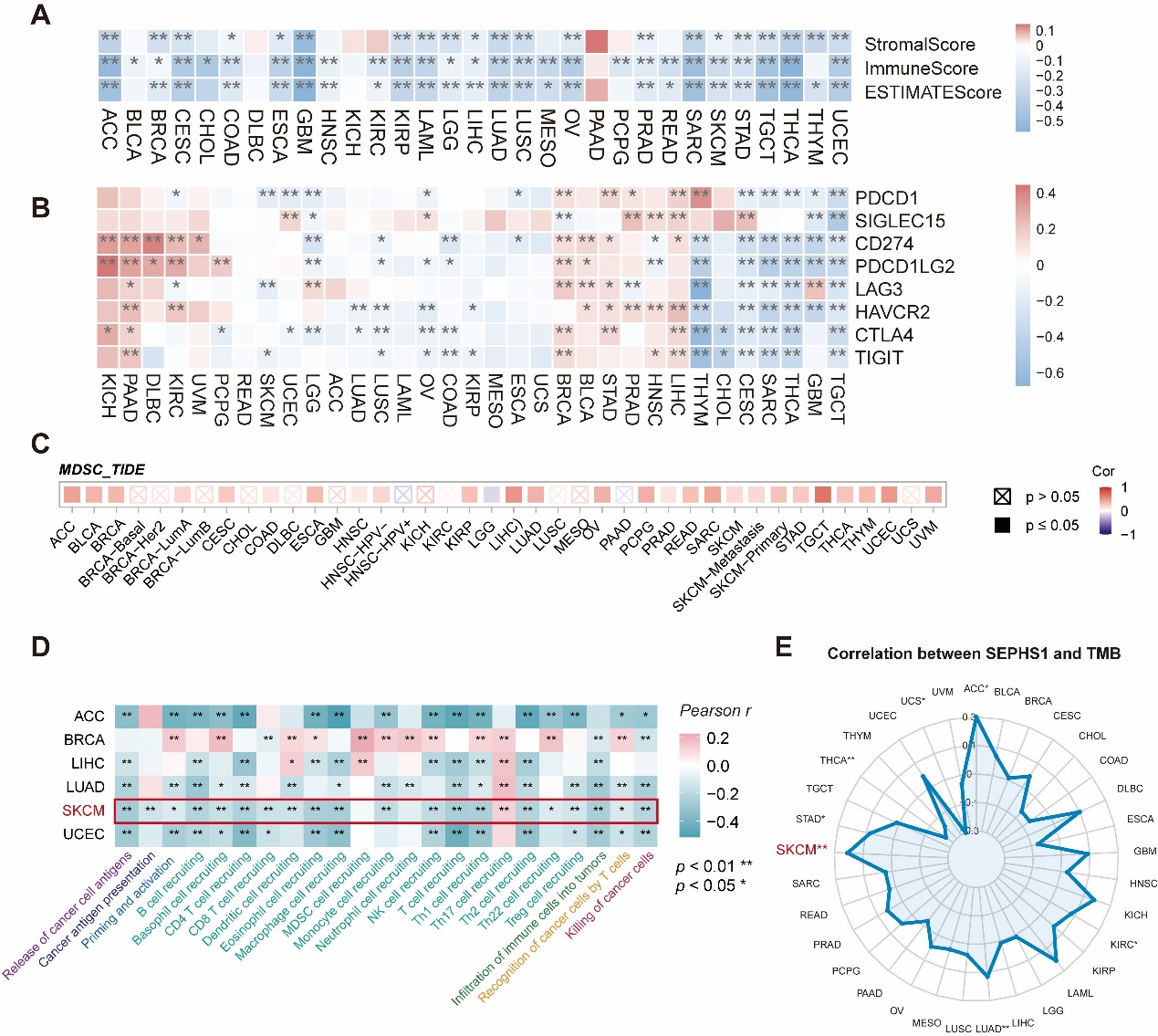
**Figure S7. SEPHS1 may contribute to malignant progression by shaping an immunosuppressive tumor microenvironment across multiple cancers.** (A) Correlation heatmap showing the associations between SEPHS1 mRNA expression and ESTIMATE score, stromal score, and immune score. Statistical significance is indicated as *****p* < 0.0001, ****p* < 0.001, ***p* < 0.01, **p* < 0.05, ns *p* ≥ 0.05. (B) Correlation heatmap showing the associations between SEPHS1 expression and mRNA levels of immune checkpoint molecules including PDCD1, CD274, PDCD1LG2, LAG3, HAVCR2, CTLA4, and TIGIT. (C) Correlation heatmap displaying the relationship between SEPHS1 expression and MDSC infiltration scores based on TIMER database data. (D) Correlation heatmap showing associations between SEPHS1 expression and tumor immune cycle signature scores in selected solid tumors. Pearson correlation coefficients were used, with *p* < 0.05 considered statistically significant. (E) Radar plot illustrating the correlations between SEPHS1 expression and tumor mutational burden across cancer types.

**
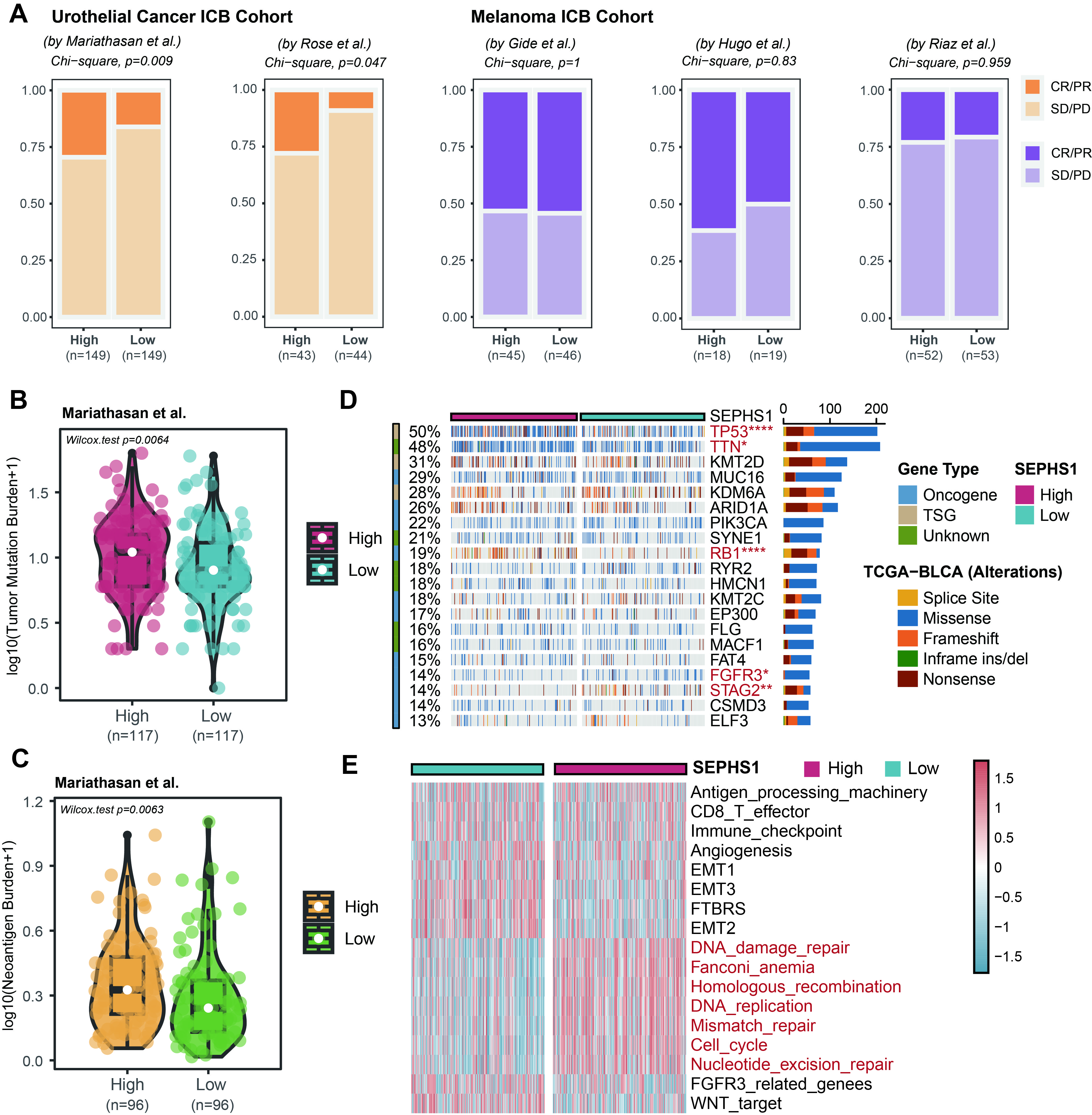
**

**Figure S8. SEPHS1 expression correlates with immune checkpoint therapy response and immunogenic features in bladder cancer.** (A) Bar plots show the proportion of patients with different clinical responses stratified by SEPHS1 expression across urothelial cancer and melanoma ICB cohorts. ICB cohorts were divided into SEPHS1 high and low expression groups according to the median expression value. Chi-square tests were used for significance assessment. (B) Violin plots compare tumor mutational burden (TMB) between high and low SEPHS1 groups in the Mariathasan cohort; statistical significance was determined by Wilcoxon rank-sum test. (C) Violin plots show the distribution of neoantigen load in high vs. low SEPHS1 groups. (D) Waterfall plot depicts the mutation landscape in high and low SEPHS1 bladder cancer samples from TCGA-BLCA. Gene types and mutation categories are annotated. (E) Heatmap shows the enrichment of immune and oncogenic pathways in SEPHS1-high and SEPHS1-low TCGA-BLCA tumors. Scores were determined using the ssGSEA method.
